# SLPI is a critical mediator that controls PTH-induced bone formation

**DOI:** 10.1038/s41467-021-22402-x

**Published:** 2021-04-09

**Authors:** Akito Morimoto, Junichi Kikuta, Keizo Nishikawa, Takao Sudo, Maki Uenaka, Masayuki Furuya, Tetsuo Hasegawa, Kunihiko Hashimoto, Hiroyuki Tsukazaki, Shigeto Seno, Akira Nakamura, Daisuke Okuzaki, Fuminori Sugihara, Akinori Ninomiya, Takeshi Yoshimura, Ryoko Takao-Kawabata, Hideo Matsuda, Masaru Ishii

**Affiliations:** 1 Department of Immunology and Cell Biology, Graduate School of Medicine and Frontier Biosciences, Osaka University, Osaka, Japan; 2 WPI-Immunology Frontier Research Center, Osaka University, Osaka, Japan; 3 Laboratory of Bioimaging and Drug Discovery, National Institutes of Biomedical Innovation, Health and Nutrition, Osaka, Japan; 4 Department of Bioinformatic Engineering, Graduate School of Information Science and Technology, Osaka University, Osaka, Japan; 5 Division of Immunology, Tohoku Medical and Pharmaceutical University, Miyagi, Japan; 6 Genome Information Research Center, Research Institute for Microbial Diseases, Osaka University, Osaka, Japan; 7 Core Instrumentation Facility, Immunology Frontier Research Center and Research Institute for Microbial Diseases, Osaka University, Osaka, Japan; 8 Medical Affairs Department, Asahi Kasei Pharma Corporation, Tokyo, Japan; 9 Laboratory for Pharmacology, Pharmaceuticals Research Center, Asahi Kasei Pharma Corporation, Tokyo, Japan

**Keywords:** Fluorescence imaging, Pharmacology, Bone

## Abstract

Osteoclastic bone resorption and osteoblastic bone formation/replenishment are closely coupled in bone metabolism. Anabolic parathyroid hormone (PTH), which is commonly used for treating osteoporosis, shifts the balance from osteoclastic to osteoblastic, although it is unclear how these cells are coordinately regulated by PTH. Here, we identify a serine protease inhibitor, secretory leukocyte protease inhibitor (SLPI), as a critical mediator that is involved in the PTH-mediated shift to the osteoblastic phase. *Slpi* is highly upregulated in osteoblasts by PTH, while genetic ablation of *Slpi* severely impairs PTH-induced bone formation. *Slpi* induction in osteoblasts enhances its differentiation, and increases osteoblast–osteoclast contact, thereby suppressing osteoclastic function. Intravital bone imaging reveals that the PTH-mediated association between osteoblasts and osteoclasts is disrupted in the absence of SLPI. Collectively, these results demonstrate that SLPI regulates the communication between osteoblasts and osteoclasts to promote PTH-induced bone anabolism.

## Abstract (web summary)

The mechanism by which parathyroid hormone mediates the switch from bone resorption to bone formation is unclear. Here, the authors show that SLPI regulates the communication between osteoblasts and osteoclasts to promote the anabolic effect of parathyroid hormone.

## Introduction

Skeletal tissue undergoes dynamic remodeling throughout the life course. Bone formation by osteoblasts and bone resorption by osteoclasts occur in spatially and temporally discrete units on the bone surface, and bone resorption is followed by bone formation^1^. The activities of osteoblasts and osteoclasts are linked by intercellular signaling for balanced bone remodeling. Given that bone formation and resorption are tightly coupled processes in the bone microenvironment, information concerning the spatiotemporal distribution of the cells involved in these processes would provide insight into bone remodeling. Previously, we visualized osteoblasts and osteoclasts by intravital multi-photon microscopy and revealed that direct contact with osteoblasts inhibited the bone-resorbing activity of osteoclasts in vivo^2^. Moreover, osteoblasts and osteoclasts were segregated in a homeostatic state, whereas intermittent parathyroid hormone (PTH) treatment dynamically altered their distribution and increased their direct cell–cell interactions, thereby inhibiting bone resorption by osteoclasts.

PTH is a major endocrine inducer and stimulator of bone remodeling. PTH (1–34) (teriparatide), a biologically active N-terminal fragment of human PTH, is used clinically to increase bone mass in osteoporosis^3,4^. PTH stimulates osteoblasts, and indirectly activates osteoclasts by regulating receptor activator of NF-κB ligand (RANKL) and osteoprotegerin in osteoblast-lineage cells^5,6^. It has been proposed that the anabolic action of PTH requires the presence of osteoclasts. In a murine model of osteopetrosis caused by a lack of osteoclasts, the anabolic effect of PTH treatment was blunted^7,8^. In the clinic, concomitant treatment of osteoporosis with alendronate, an inhibitor of osteoclasts, significantly decreased the anabolic response to PTH^9,10^. These results imply that the shift from osteoclasts to osteoblasts contributes to the osteoanabolic effect of PTH^11^. However, the mechanisms that coordinately regulate these two cell types in PTH anabolism are unknown.

Secretory leukocyte protease inhibitor (SLPI) is a serine protease inhibitor, secreted in response to tissue damage that plays multiple roles in inflammation in vivo. SLPI inhibits inflammation and initiates the healing of damaged tissue^12^. These functions are dependent on its protease inhibition^13,14^ and transcriptional regulation^15^. Therefore, SLPI serves as a molecular switch from inflammation to tissue proliferation^16^.

Although expression of SLPI in osteoblasts has been reported^17^, the functional role of SLPI in bone metabolism in vivo, particularly in bone resorption, has not been described. In the present study, we found that PTH strongly induced *Slpi* expression in osteoblasts, and the effect of PTH on bone was dependent on SLPI. Moreover, SLPI expressed by osteoblasts promoted direct cell–cell contact, thereby shifting the balance from osteoclastic activity to osteoblastic activity. Therefore, SLPI is an essential mediator that can promote a PTH-mediated switch from bone resorption to bone formation.

## Results

### Osteoblast Slpi expression is induced by PTH in vivo

The anabolic effect of PTH depends on its binding to the PTH receptor (PTH1R) on osteoblast-lineage cells^11^. Three weeks of PTH administration induced merged distribution of osteoblasts and osteoclasts (Supplementary Fig. S1a). In addition, the responses of osteoblasts and osteoclasts to PTH treatment in femoral trabecular bone were compatible to those in their calvarial counterparts (Supplementary Fig. S1b). To assess gene expression changes in response to PTH, we first isolated osteoblasts with high purity from adult bone tissues of transgenic mice, in which mature osteoblasts specifically express enhanced cyan fluorescent protein (ECFP) driven by the type I collagen promoter (i.e., Col2.3-ECFP mice)^2^. Using flow cytometry, we detected the lineage (Lin^−^) CD45^−^ ECFP^+^ after sequential enzymatic digestion, but not from non-adherent bone marrow cells (Supplementary Fig. S1c). The ECFP^+^ cells expressed high levels of *Sp7* and *Bglap*, and so were identified as osteoblasts (Supplementary Fig. S1d).

We next evaluated transcripts in osteoblasts by RNA-Sequencing (RNA-Seq), using a PTH mouse model^18^ treated intermittently with PTH or vehicle for 3 weeks. We considered genes showing a twofold or greater change to be significantly differentially expressed (*p* < 0.05); 405 genes were upregulated by PTH, while 883 were downregulated. The expression levels of the osteoblast differentiation markers *Sp7* and *Bglap* were not changed (Supplementary Fig. S1e), and thus were not affected by the PTH-induced change in cell population. The mRNA level of *Fgf2*, an important growth factor for PTH anabolism^19^, significantly increased (Supplementary Fig. S1f, h).

**Fig. 1:**
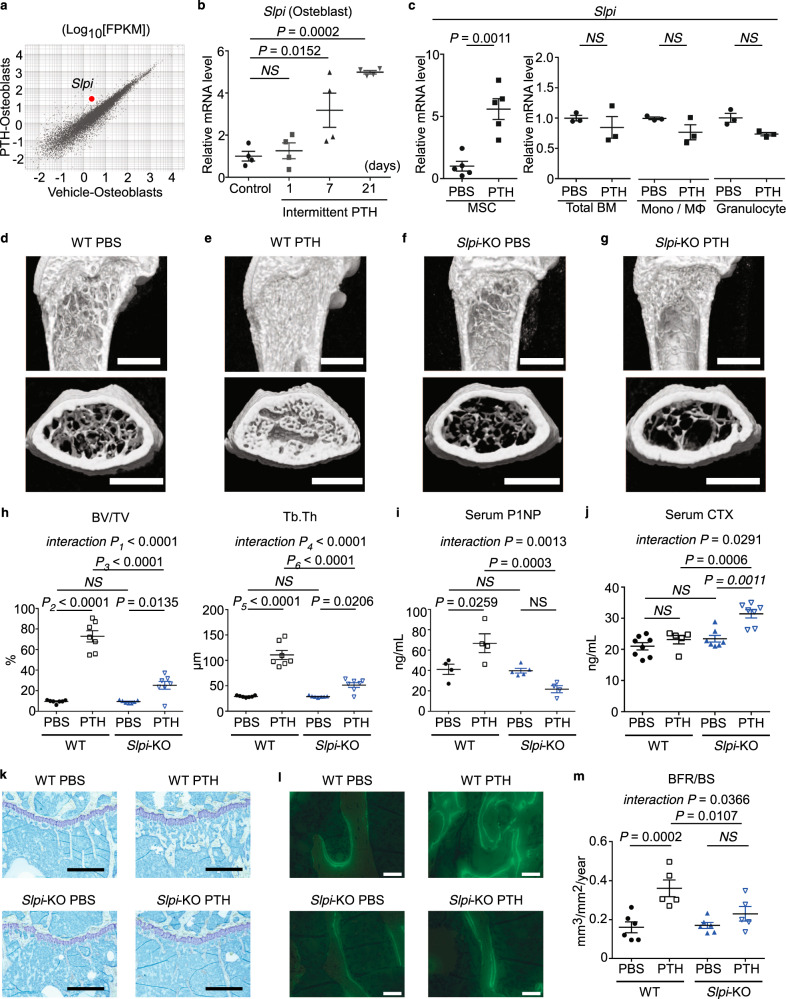
Secretory leukocyte protease inhibitor (SLPI) controls the parathyroid hormone (PTH)-induced increase in bone mass. **a** RNA-sequencing (RNA-Seq) scatter plot of differential expression between vehicle-treated osteoblasts and intermittent parathyroid hormone (PTH)-treated osteoblasts (log_10_-transformed fragments per kilobase of transcript per million mapped reads [FPKM]). **b** mRNA levels of *Slpi* in 7AAD^−^ Lin^−^ CD45^−^ ECFP^+^ osteoblasts as determined by quantitative polymerase chain reaction (qPCR) analysis (*n* = 4 biologically independent samples per group). **c** mRNA levels of *Slpi* as determined by qPCR analysis (*n* = 5 mice per group for the analysis of MSC, and *n* = 3 mice per group for the analysis of total BM, Mono/Mφ, and granulocytes). MSC, mesenchymal stem cells (Lin^−^ CD45^−^ CD31^−^ Sca1^+^ CD51^+^ population); BM, total bone marrow cells; Mono/Mφ, monocyte/macrophage (CD45^+^ Ly6G^+^ population); granulocytes (CD45^+^ Ly6G^−^ F4/80^+^ population). **d–g** Representative micro-computed tomography images of the femurs. Scale bar, 1000 µm. **h** Ratio of bone volume to total bone volume (BV/TV), and trabecular thickness (Tb.Th) values (*n* = 7 mice per group). **i** Serum type I procollagen N-terminal propeptide (P1NP) levels, as measured by enzyme-linked immunosorbent assay (ELISA) (WT PBS: *n* = 4 mice, WT PTH: *n* = 4 mice, *Slpi*-KO PBS: *n* = 5 mice, *Slpi*-KO PTH: *n* = 4 mice). **j** Serum type I procollagen C-terminal telopeptide (CTX) levels, as measured by ELISA (WT PBS: *n* = 8 mice, WT PTH: *n* = 5 mice, *Slpi*-KO PBS: *n* = 7 mice, *Slpi*-KO PTH: *n* = 7 mice). **k** Histological analysis of the proximal tibias. Images are toluidine blue-stained sections (Representative image, *n* = 3 biologically independent experiments). Scale bars, 500 µm. **l** Merged image of the calcein band and bright field of the proximal tibias (Representative image, *n* = 3 biologically independent experiments). Scale bar, 50 µm. **m** Bone formation rate (BFR/BS) values (WT PBS: *n* = 6 mice, WT PTH: *n* = 5 mice, *Slpi*-KO PBS: *n* = 6 mice, *Slpi*-KO PTH: *n* = 5 mice). For details and statistical analysis, see Table S2. Data are means ± SEM. NS, not significant. Statistical significance was determined by ANOVA with Dunnett’s test (**b**), Tukey’s test (**h**, **i**, **j**), Šidák’s test (**m**), and two-tailed Student’s *t*-test (**c**). **h** *P*_1_ exact value = 2.8E^−7^, *P*_2_ exact value = 8.5E^−12^, *P*_3_ exact value = 2.8E^−9^, *P*_4_ exact value = 5.1E^−6^, *P*_5_ exact value = 2.0E^−10^, *P*_6_ exact value = 9.5E^−8^.

In the Gene Ontology (GO) enrichment analysis, many of the upregulated GO pathways were related to the regulation of proteolysis (Supplementary Fig. S1g). Among such genes, *Slpi* was the highly-ranked upregulated gene induced by PTH (Fig. 1a; Supplementary Fig. S1h). Quantitative polymerase chain reaction (qPCR) confirmed that PTH upregulated the expression of *Slpi* in osteoblasts in vivo (Supplementary Fig. S1i). We analyzed the time-course of the effect of PTH and found that to induce an increase in the *Slpi* mRNA levels required 1–3 weeks of repetitive injection (Fig. 1b). PTH can have anabolic or catabolic effects, depending on its mode of administration: the anabolic effect occurs by intermittent administration of PTH, but not by continuous infusion^20^. *Slpi* upregulation was not induced by continuous infusion of PTH for 3 weeks (a catabolic regimen; Supplementary Fig. S1j).

We also analyzed *Slpi* expression in other cell populations in bone tissues. We examined the cell populations reported to express *Slpi* in an online database (Gene Expression Commons^21^) (Supplementary Fig. S2). qPCR analyses revealed that PTH treatment increased *Slpi* expression in Lin^−^CD45^−^CD31^−^CD51^+^Sca1^+^ mesenchymal stem cells (MSCs)^22^ (Fig. 1c), but not in total bone marrow cells, CD45^+^Ly6G^−^F4/80^high^ bone marrow monocytes/macrophages, or CD45^+^Ly6G^+^ bone marrow granulocytes (Fig. 1c). Therefore, *Slpi* was specifically and highly upregulated in osteoblast-lineage cells by repetitive PTH injection in vivo.

### SLPI controls the PTH-induced increase in bone mass

The above results prompted us to explore the importance of SLPI in physiological bone remodeling in vivo. Initially, we evaluated baseline bone modeling and remodeling in *Slpi*-knockout (KO) mice^23^. In a homeostatic state, 5-week-old female mice were injected with vehicle for 6 weeks; distal femoral metaphysis was analyzed by micro-computed tomography (µCT) when the mice were 11 weeks of age. *Slpi*-KO mice displayed no overt trabecular bone phenotype, compared with wild-type (WT) mice (Fig. 1d, f, h; Supplementary Movies 1, 3). In vitro, WT and *Slpi*-KO primary osteoblasts showed identical bone-forming potential, as demonstrated by Alizarin Red S staining (Supplementary Fig. S3a). In addition, the numbers of osteoclasts differentiated from *Slpi*-KO macrophages were similar to the numbers differentiated from WT macrophages (Supplementary Fig. S3b). Together, these data suggest that *Slpi*-KO mice in a homeostatic state show normal bone remodeling with no aberrant phenotype.

To test the function of *Slpi* expression in PTH anabolism, we next examined the effect of PTH on *Slpi*-KO mice. Eleven-week-old WT mice subjected to intermittent PTH treatment for 6 weeks showed a significant increase in trabecular content, compared with vehicle-treated mice (Fig. 1d, e; Supplementary Movies 1, 2). This was apparent from the cancellous bone volume (BV/TV), and trabecular thickness (Tb.Th) (Fig. 1h). In contrast, the bone anabolic effect of PTH was severely disrupted in *Slpi*-KO mice; specifically, the rate of increase in BV/TV in *Slpi*-KO mice was less than half that in WT mice (Fig. 1e, g, h; Supplementary Movies 2, 4). Similarly, PTH-treated WT mice had a significantly higher serum levels of type I procollagen N-terminal propeptide (P1NP), a biochemical marker of bone formation, compared to vehicle-treated WT control mice, which was disrupted in *Slpi*-KO mice (Fig. 1i). Assessment of the serum levels of C-terminal telopeptide of collagen (CTX), a biochemical marker of resorption, revealed that intermittent PTH treatment did not increase bone resorption in WT mice, but did increase it in *Slpi*-KO mice (Fig. 1j). Therefore, SLPI affects both bone formation and bone resorption in PTH anabolism, which results in significantly different trabecular bone phenotypes in the two genotypes. In addition, we analyzed trabecular bone in male mice to investigate the effect of sex on PTH anabolism. Five-week-old male mice were injected with vehicle or PTH for 6 weeks, and distal femoral metaphysis was analyzed by µCT. The effect of PTH was weaker in WT male mice than in WT female mice, while PTH treatment decreased trabecular bone mass in *Slpi*-KO male mice (Supplementary Fig. S4). Therefore, these data demonstrate that the anabolic effect of PTH is also dependent on SLPI in male mice.

Given that the anabolic action of PTH results from enhanced bone formation, we examined the effect of ablation of *Slpi* on the cellular response to PTH via histomorphometric analysis. Twelve-week-old female WT or *Slpi*-KO mice were treated daily with vehicle or PTH for 4 weeks and their proximal tibias were analyzed. The effects of PTH treatment on bone formation rate (BFR/BS) were significantly reduced in *Slpi*-KO mice compared with WT controls (Fig. 1k–m; Supplementary Table S1). Collectively, bone histomorphometric analysis supported the notion that PTH-induced bone formation is impaired in *Slpi*-KO mice, consistent with the microstructural and bone metabolic marker analyses.

### PTH regulates Slpi expression via PKA and Erk signaling

Next, we evaluated whether *Slpi* expression in osteoblasts is directly regulated by PTH in vitro. In cultures of MC3T3-E1 cells, a clonal osteoblastic cell line, PTH increased *Slpi* expression levels at the peak between 6 and 12 h after stimulation (Supplementary Fig. S5a). PTH1R is a seven G protein-coupled transmembrane protein expressed in osteoblast-lineage cells. In general, activated PTH1R selectively couples several G-protein subclasses, activating the cAMP dependent protein kinase A (PKA), intracellular Ca2^+^, protein kinase C (PKC)^24^, and β-arrestin-ERK1/2^25^ intracellular signaling pathways. The PKA activator forskolin markedly increased *Slpi* expression in MC3T3-E1 cells, while the specific PKC activator phorbol 12-myristate 13-acetate (PMA), and the calcium ionophore A23187, did not upregulate *Slpi* (Supplementary Fig. S5b). Consistent with these findings, two pharmacological PKA inhibitors, H-89 and Rp-adenosine-3′,5′-cyclic monophosphorothioate (Rp-cAMPS), inhibited the *Slpi* expression induced by PTH (Supplementary Fig. S5c). In addition, the pharmacological MEK inhibitor U0126 inhibited the PTH-induced upregulation of *Slpi* (Supplementary Fig. S5c). Therefore, PTH activates the cAMP/PKA and β-arrestin-ERK1/2 signaling pathways, which mediates the effect of PTH1R signaling on *Slpi* transcription.

### Induction of Slpi expression enhances osteoblast differentiation and proliferation

**Fig. 2:**
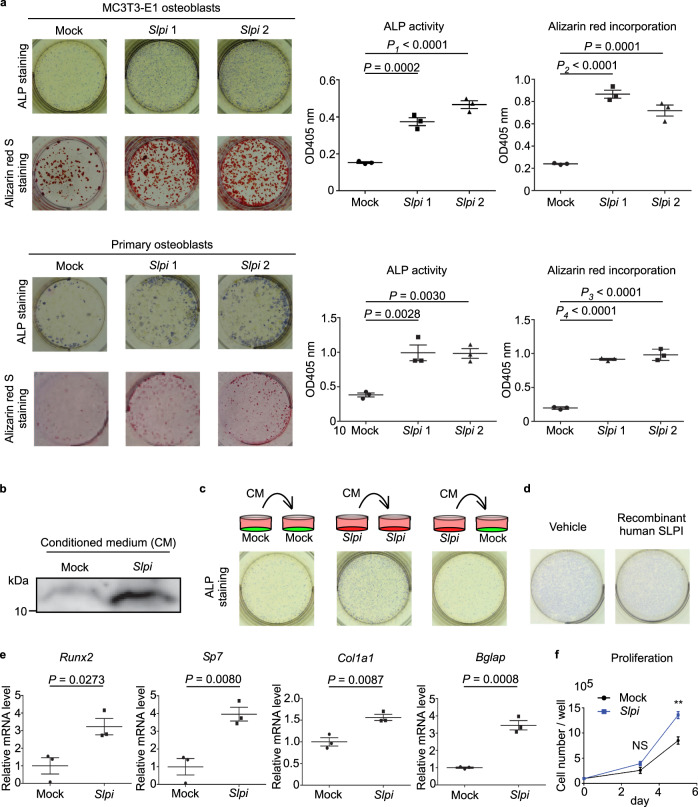
Induction of *Slpi* enhances osteoblast differentiation and proliferation. **a** Representative images of MC3T3-E1 and primary mouse calvarial osteoblasts transfected with either pMX-*Slpi*-IRES-puro or pMX-IRES-puro by alkaline phosphatase (ALP) staining (upper) and Alizarin Red S staining (lower) (*n* = 3 biological replicates per group). β-glycerophosphate was used at a concentration of 10 mM. **b**, Immunoblot of SLPI in conditioned medium from control MC3T3-E1 and *Slpi*-overexpressing cells (Representative blot, *n* = 2 biologically independent experiments). **c** Typical images of ALP staining of MC3T3-E1 cells. Control (mock) or *Slpi* overexpressing (Slpi) MC3T3-E1 cells were treated with mock or *Slpi* conditioned medium under osteogenic conditions for 3 days. **d** ALP-stained images of MC3T3-E1 cells treated with vehicle or human recombinant SLPI (10 µg/mL) under osteogenic conditions for 3 days. **e** qPCR analysis of MC3T3-E1 cells transfected with pMX-*Slpi*-IRES-puro or pMX-IRES-puro (*n* = 3 biological replicates per group). Cells were cultured in osteogenic differentiation medium for 3 days, and the expression levels of osteoblast transcript regulators (*Runx2*, *Sp7*) and differentiation marker genes (*Bglap*, *Col1a1*) were quantified. **f** Proliferation of MC3T3-E1 cells transfected with pMX-*Slpi*-IRES-puro or pMX-IRES-puro. Cells were cultured in α- MEM for 72 or 120 h after adherence (*n* = 3 biological replicates per group). **P = 0.0071. Data are means ± SEM. NS, not significant. Statistical significance was determined by ANOVA with Dunnett’s test (**a**), and two-tailed Student’s *t*-test (**e**, **f**). **a** *P*_*1*_ approximate value < E^−15^*, P*_*2*_ approximate value < E^−15^*, P*_*3*_ approximate value < E^−15^*, and P*_*4*_ approximate value < E^−15^.

To examine the biological role of SLPI in osteoblasts, we constructed a retrovirus vector for constitutive *Slpi* expression (pMX-*Slpi*-IRES-Puro), which was engineered to express both *Slpi* and a puromycin-resistance gene. Induction of *Slpi* with pMX-*Slpi*-IRES-Puro enhanced alkaline phosphatase (ALP) activity, and accelerated calcification in MC3T3-E1 cells and primary osteoblasts, as determined by Alizarin Red S staining (Fig. 2a). As SLPI is secreted from cells into cervical mucus or bronchial and nasal secretions^26^, MC3T3-E1 cells also secreted SLPI into culture medium (Fig. 2b). To examine an autocrine effect of SLPI, we added conditioned medium from *Slpi*-overexpressing MC3T3-E1 cells to control (mock) cells. The results showed that conditioned medium from *Slpi*-overexpressing cells had no effect on osteoblast differentiation (Fig. 2c). Consistently, conditioned medium from *Slpi*-overexpressing cells did not upregulate *Runx2* (Cbfa1/AML3), the osteogenic master gene for bone formation^27,28^, the osteoblast-specific transcription factor Osterix (*Sp7*), or the early osteoblast marker gene *Col1a1* (Supplementary Fig. S6a). The late osteoblast marker gene osteocalcin (*Bglap*) was slightly increased by treatment with conditioned medium (Supplementary Fig. S6a). The addition of recombinant human SLPI to the culture medium also did not enhance ALP activity in MC3T3-E1 cells (Fig. 2d). We confirmed that overexpression of *Slpi* upregulated *Runx2, Sp7*, *Col1a1*, and *Bglap* in MC3T3-E1 cells (Fig. 2e; Supplementary Fig. S6b).

Next, to investigate the role of SLPI in osteoblasts, we analyzed its effects on intracellular proteins. Silver staining of intracellular proteins from MC3T3-E1 osteoblasts showed multiple enhanced bands in *Slpi*-overexpressing cells (Supplementary Fig. S6c). These protein extracts were analyzed by liquid chromatography tandem mass spectrometry (timsTOF pro, Bruker) and the results showed that the levels of 694 proteins were increased, while the levels of 131 proteins were decreased, by SLPI in MC3T3-E1 osteoblasts. Although SLPI is a regulator of IκB^13,29^ and proepithelin^16^ degradation, both of which activate osteoblasts^30,31^, the levels of these proteins did not increase. Four proteins detected by mass spectrometry analysis were associated with “positive regulation of osteoblast differentiation,” according to NCBI GO annotations. Of those proteins, catenin beta-1 (β-catenin) was upregulated by SLPI, and higher levels of β-catenin protein were detected by immunoblotting in *Slpi*-overexpressing cells (Supplementary Fig. S6d). Consistent with regulation of intracellular β-catenin levels by the proteasomal degradation pathway, the mRNA levels of β-catenin were not increased by SLPI (Supplementary Fig. S6e). Therefore, canonical Wnt signaling is a candidate downstream target of SLPI, although the mechanistic relationship between SLPI and the ubiquitin-proteasome pathway is unclear.

Because osteoblast proliferation is also an important indicator of osteogenesis, we analyzed the proliferation of cultured MC3T3-E1 cells. Compared with mock MC3T3-E1 cells, the numbers of *Slpi*-overexpressing cells began to increase at day 3; the difference was statistically significant at day 5 (Fig. 2f), indicating that SLPI promotes osteoblast proliferation. The anabolic response of osteoblasts to PTH is in part due to reduced apoptosis of osteoblasts and their precursors^32^. Therefore, we evaluated the antiapoptotic effect of SLPI on MC3T3-E1 cells by flow cytometry. Neither 7AAD^−^ annexin V^+^ (early apoptotic) cell counts nor 7AAD^+^ annexin V^+^ (late apoptotic) cell counts were increased by *Slpi* overexpression (Supplementary Fig. S6f).

The above findings show that SLPI promotes osteoblast proliferation and differentiation by targeting key transcription factors such as β-catenin, Runx2, and Osterix.

### SLPI is associated with adhesion between osteoblasts and osteoclasts

**Fig. 3:**
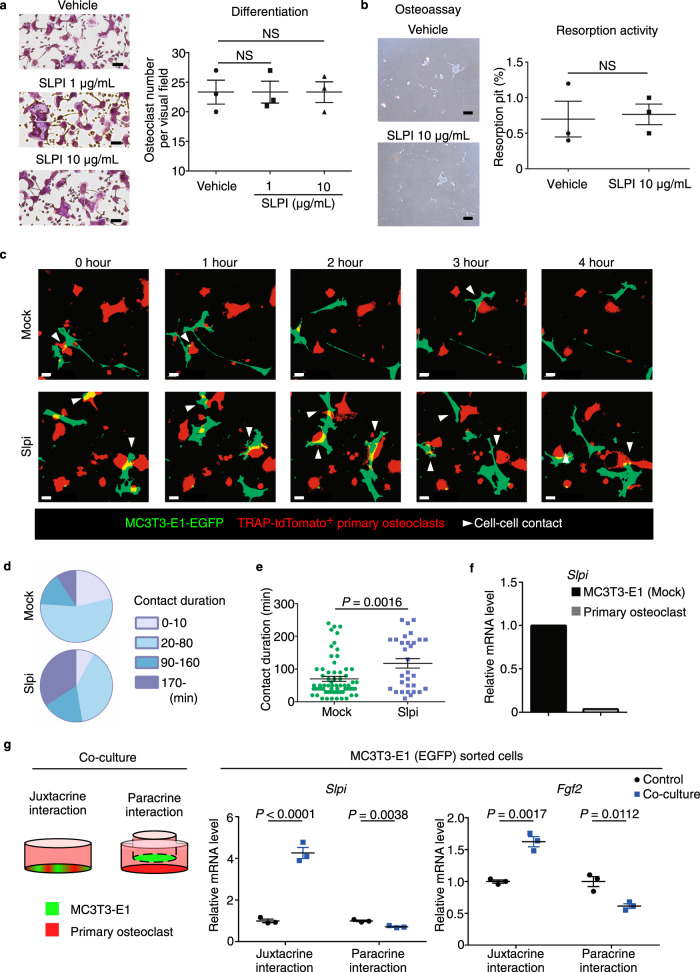
SLPI is associated with adhesion between osteoblasts and osteoclasts. **a** TRAP-stained cells showing the effect of recombinant human SLPI on osteoclastogenesis. Scale bar, 100 µm. Right, numbers of TRAP^+^ cells (*n* = 3 biological replicates per group). **b** Effects of SLPI on the generation of bone resorption pits. Mature osteoclasts were transferred to a Corning Osteoassay Surface in the presence or absence of recombinant human SLPI. After 48 h, cells were removed, and resorption pits were visualized. Scale bar, 200 µm. Right, area of resorption pits (%) (*n* = 3 biological replicates per group). **c** Fluorescence images of mock (upper panel) or *Slpi*-overexpressing (lower panel) MC3T3-E1-EGFP cells in contact with TRAP-tdTomato^+^ primary osteoclasts (Representative image, *n* = 3 biologically independent experiments). Confocal fluorescence microscopy images were acquired at 0, 120, 240, 360, and 480 min. White triangles, direct cell–cell contact. Scale bar, 10 µm. **d**, **e** Analysis of osteoblast–osteoclast contact duration based on the data in **c** (Mock: *n* = 67 cells examined over six independent experiments, *Slpi*: *n* = 31 cells examined over 5 independent experiments). **f** Relative expression levels of *Slpi* in enhanced green fluorescent protein (EGFP)-labeled MC3T3-E1 cells and tdTomato-labeled osteoclasts. After coculture, MC3T3-E1 cells and osteoclasts were separated (*n* = 1 experiment per group). **g** Left, diagrammatic representation of in vitro coculture of osteoclasts with MC3T3-E1-EGFP cells. Right panel shows the effects of coculture on *Slpi* and *Fgf2* expression in MC3T3-E1-EGFP cells (*n* = 3 biological replicates per group). Data are means ± SEM. NS, not significant. Statistical significance was determined by ANOVA with Dunnett’s test (**a**), and two-tailed Student’s *t*-test (**b**, **e**, **g**).

To determine how SLPI affects bone resorption, we explored the paracrine role of SLPI in signaling between osteoblasts and osteoclasts. Because SLPI is a regulator of nuclear factor kappa B (NF-κB) in monocyte-lineage cells, we examined whether osteoclast differentiation via NF-κB signaling^33^ was modulated by SLPI secreted from osteoblast-lineage cells. Treatment with recombinant human SLPI did not affect RANKL-induced osteoclast differentiation in vitro (Fig. 3a). Osteoclastic bone degradation is mediated by cysteine proteases and matrix metalloproteinases, which are not considered mechanistic targets of the serine proteinase inhibitor SLPI^34–36^. Consistent with this, we found that osteoclast pit-forming activity in vitro was not affected by recombinant human SLPI (Fig. 3b). Therefore, SLPI secreted from osteoblasts does not directly affect osteoclast differentiation or bone-resorbing activity.

Proteases and protease inhibitors are associated with cellular detachment from surrounding components. Indeed, SLPI in osteoblasts is reportedly associated with focal cell adhesion^37^. Therefore, we examined the effect of SLPI on adhesion between osteoblasts and osteoclasts by coculture experiments. When control (mock) MC3T3-E1 cells were cultured with primary osteoclasts, the duration of osteoblast-osteoclast colocalization was generally less than 1 h (Fig. 3c, d; Supplementary Movie 5). In contrast, induction of *Slpi* in MC3T3-E1 cells slightly increased the ratio of long cell-cell contact (Fig. 3c, d; Supplementary Movie 6). Consistent with these findings, quantitative analysis revealed that contact duration was significantly increased by *Slpi* induction (Fig. 3e). Also, *Slpi* mRNA was expressed in EGFP^+^ osteoblastic cells, but not in tdTomato^+^ osteoclasts, in coculture experiments (Fig. 3f).

Given that SLPI promotes leukocyte chemotaxis in vitro^38^, we examined whether SLPI secreted from osteoblasts directly attracts osteoclasts. We evaluated the effects of SLPI inhibition by commercially available neutralizing antibodies on osteoclast mobility. Time-lapse imaging was performed to assess cocultures of MC3T3-E1 cells and primary osteoclasts until 12 h after treatment with an SLPI neutralizing antibody or an IgG isotype-matched control antibody. However, treatment with an SLPI neutralizing antibody did not inhibit the osteoblast–osteoclast contact enhanced by SLPI (Supplementary Fig. S7).

Next, to investigate the molecular mechanisms by which *Slpi* induces colocalization of osteoblasts with osteoclasts further, we performed an RNA-Seq analysis using control (mock) MC3T3-E1 cells and *Slpi*-overexpressing cells (Supplementary Fig. S8a). Chemokines and growth factors have key roles in physiological bone remodeling by controlling migration, and localization of osteoclasts and their precursors^39,40^. However, these factors were overall not remarkably changed by SLPI in osteoblasts (Supplementary Fig. S8a). Similarly, we did not observe reduced expression of the repulsive guidance factor Plexin-B1, which is known to contribute to the spatial segregation of osteoblasts and osteoclasts^41,42^ (Supplementary Fig. S8a).

Notably, among the canonical pathways affected by SLPI, Wnt/β-catenin signaling was one of the most significantly affected (Supplementary Fig. S8b), consistent with the regulation of β-catenin by SLPI (Supplementary Fig. 6d). RhoA signaling was another pathway significantly affected by SLPI (Supplementary Fig. S8b). Rho-associated coiled-coil kinases (ROCKs) are pivotal downstream effectors of RhoA. Immunoblot analysis revealed that ROCK2, but not ROCK1, was upregulated at the protein level in *Slpi*-overexpressing cells (Supplementary Fig. S8c). ROCK inhibitor Y-27632 significantly prevented the osteoclast-osteoblast contact that had been promoted by SLPI, suggesting that ROCK is associated with *Slpi*-induced osteoblast-osteoclast adhesion (Supplementary Fig. S8d). Moreover, *Slpi*-overexpressing MC3T3-E1 cells had comparably enhanced transmembrane glycoprotein ICAM-1 expression (Supplementary Fig. S8e). These results were consistent with previous studies showing that RhoA signaling is associated with heterotypic cell–cell adhesion, such as hematopoietic stem cell-niche cell adhesion^43^, and osteoblast–osteoclast adhesion^44^, through the expression of transmembrane cell adhesion molecules^44,45^.

Next, we analyzed how interactions between osteoblasts and osteoclasts influenced the activities of both cell types. With respect to bone formation, coculture of osteoblasts and osteoclasts reportedly promoted mineralization in osteoblasts^46^. Consistent with this observation, the presence of differentiated osteoclasts led to markedly increased ALP activity in primary osteoblasts (Supplementary Fig. S9a, b). Alizarin Red S staining revealed that the presence of mature osteoclasts led to markedly increased mineralization in primary osteoblasts (Supplementary Fig. S9a, b). Notably, direct coculture with mature osteoclasts for 24 h significantly increased *Slpi* expression in MC3T3-E1 cells (juxtacrine interaction in Fig. 3g). In addition, the expression of *Fgf2*, which was upregulated in PTH-treated osteoblasts, was increased in the presence of osteoclasts. We also performed experiments using the Transwell system, which enables sharing of culture medium between osteoblasts and osteoclasts without direct contact. However, *Slpi* and *Fgf2* expression in osteoblasts did not increase under this condition (paracrine interaction in Fig. 3g), suggesting that direct osteoblast–osteoclast contact plays a role in increasing the levels of these anabolic factors. With respect to bone resorption, the presence of differentiated MC3T3-E1 cells inhibited bone marrow macrophages from undergoing osteoclastogenesis (Supplementary Fig. S9c). Resorption pit formation by mature osteoclasts was also reduced during coculture with differentiated MC3T3-E1 cells (Supplementary Fig. S9d). Collectively, these results suggest that the close contact between osteoblasts and osteoclasts would result in increased bone formation and decreased bone resorption. Therefore, SLPI-induced colocalization presumably promotes interactions between osteoblasts and osteoclasts, modifying the activities of both cell types to facilitate the anabolic action of PTH.

### The PTH-induced direct association between osteoblasts and osteoclasts was disrupted in the absence of Slpi

**Fig. 4:**
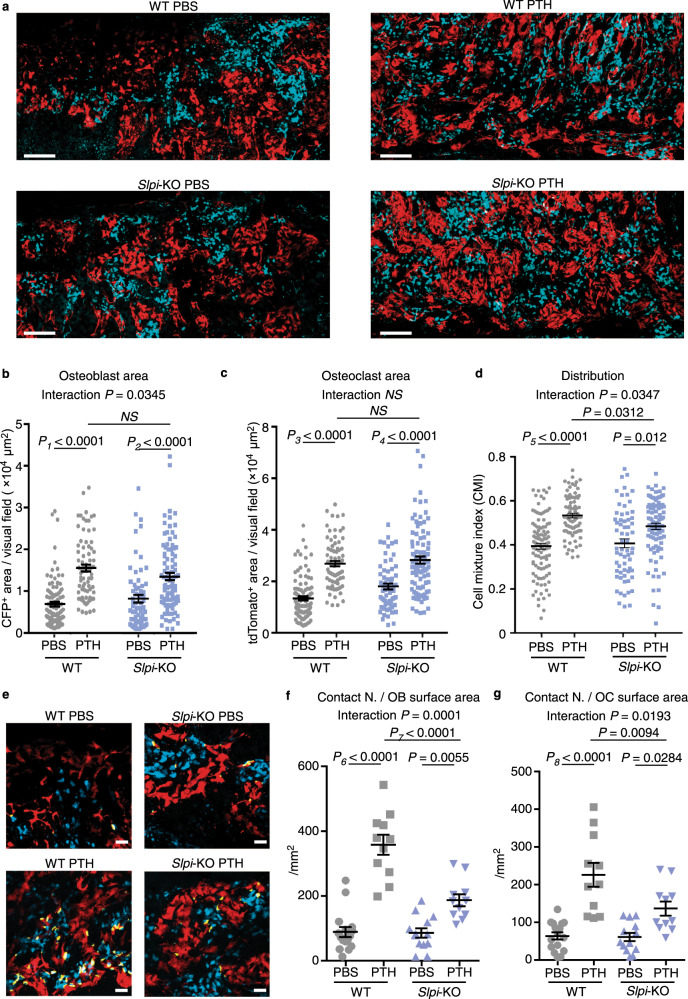
The PTH-induced direct association between osteoblasts and osteoclasts was disrupted in the absence of SLPI. **a** Maximum-intensity projection (MIP) images of skull tissues obtained by intravital microscopy (Representative image, *n* = 6 biologically independent experiments). Left panel, tiling image of Col2.3-ECFP/TRAP-tdTomato/*Slpi*^WT/WT^ mice; right panel, image of Col2.3-ECFP/TRAP-tdTomato/*Slpi*^KO/KO^ mice at 3 weeks after intermittent PBS (upper panel) or PTH (lower panel) treatment. Cyan, mature osteoblasts; red, mature osteoclasts. Scale bar, 200 µm. **b** Areas of ECFP^+^ mature osteoblasts per visual field in the tiling image. Cyan^+^ (mature osteoblast) areas were binarized using Otsu’s thresholding method. **c** Areas of tdTomato^+^ mature osteoclasts per visual field in the tiling image. tdTomato^+^ (mature osteoclast) areas were binarized using Otsu’s thresholding method. **d** Cell mixture index (CMI) values per visual field in WT mice. **b**–**d** WT PBS: *n* = 103 images from six mice, WT PTH: 80 images from five mice, *Slpi*-KO PBS: 64 images from five mice, *Slpi*-KO PTH: 93 images from six mice. **e**, MIP images of 3D colocalization. Left panel, Col2.3-ECFP/TRAP-tdTomato/*Slpi*^WT/WT^ mice; right panel, Col2.3-ECFP/TRAP-tdTomato/*Slpi*^KO/KO^ mice, at 3 weeks after intermittent PBS (upper panel) or PTH (lower panel) treatment (Representative image, *n* = 6 biologically independent experiments). Cyan, mature osteoblasts; red, mature osteoclasts. Contact areas (yellow) were defined as the areas of osteoblast and osteoclast colocalization. Scale bar, 200 µm.Numbers of contacts between osteoblasts and osteoclasts; data were normalized to the surface area of ECFP^+^ osteoblasts (**f**), and tdTomato^+^ osteoclasts (**g**), respectively (WT PBS: *n* = 11 mice, WT PTH: 11 mice, *Slpi*-KO PBS: 16 mice, *Slpi*-KO PTH: 13 mice). Data are means ± SEM. NS, not significant. Statistical significance was determined by ANOVA with Tukey’s test (**b**, **c**, **f**, **g**), and by Welch’s ANOVA followed by Bonferroni’s multiple comparisons test (**d**). **b** *P*_*1*_ exact value = 3.4E^−13^, *P*_*2*_ exact value = 9.6E^−9^. **c** *P*_*3*_ exact value = 4.3E^−13^, *P*_*4*_ exact value = 4.9E^−5^. **d** *P*_*5*_ approximate value < E^−15^. **f** *P*_*6*_ exact value = 2.1E^−12^, *P*_*7*_ exact value = 4.2E^−6^. **g** *P*_*8*_ exact value = 2.5E^−7^.

Cell-to-cell attraction and cell stickiness promote the intermingling of different cell types, whereas tension between cells results in their segregation^47^. Therefore, we used intravital multi-photon microscopy to determine whether SLPI-mediated heterophilic interaction impacted PTH-induced bone cell distribution in vivo. We developed a system enabling simultaneous visualization of osteoblasts and osteoclasts using double transgenic Col2.3-ECFP/tartrate-resistant acid phosphatase (TRAP)-tdTomato reporter mice^2^. We crossed these mice with *Slpi*-KO mice, and compared the living bone tissue of anabolic PTH-treated mice. ECFP^+^ osteoblasts and tdTomato^+^ osteoclasts were increased after 3 weeks of PTH treatment (Fig. 4a–c). The densities of ECFP^+^ osteoblasts and tdTomato^+^ osteoclasts between *Slpi*-KO and WT mice were similar after PTH treatment (Fig. 4a–c). However, the degrees of osteoblast and osteoclast gathering both increased, and their intermingling was disrupted by knockout of *Slpi*. Quantification of the cell mixture index (CMI), a cell mixture parameter calculated mathematically by hierarchical clustering^2^, demonstrated that the intermingling enhanced by PTH was partially but significantly disrupted in *Slpi*-KO mice, compared to WT mice (Fig. 4a, d; Supplementary Fig. S10). Consistent with this, the number of dynamic osteoblast–osteoclast contact events normalized to the surface area of osteoblasts or osteoclasts was significantly decreased in *Slpi*-KO mice, according to three-dimensional co-localization analysis of time-lapse images (Fig. 4e–g). These results demonstrate that SLPI changes bone cell distribution, resulting in altered cell–cell communication in the setting of PTH anabolism in vivo.

## Discussion

**Fig. 5:**
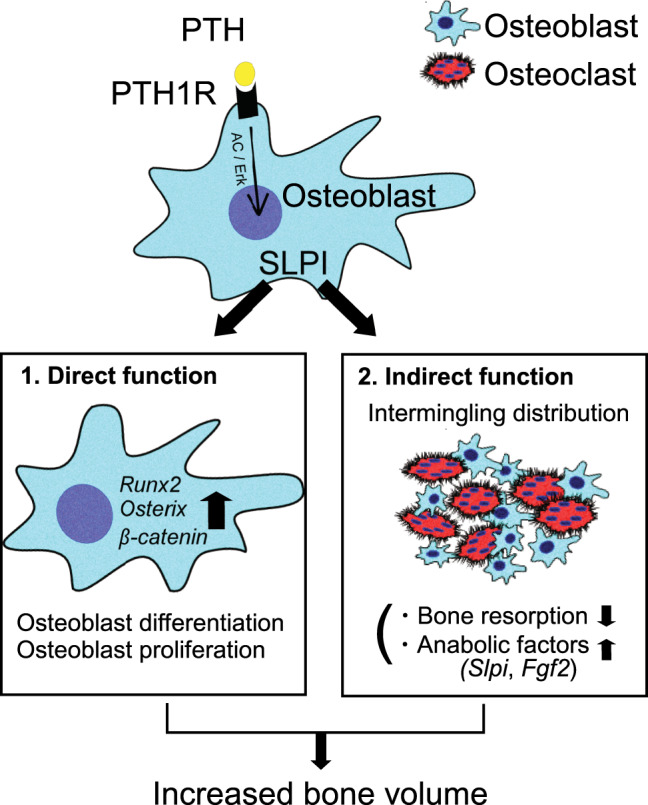
Model of SLPI functions in bone metabolism. Parathyroid hormone (PTH) binding to parathyroid hormone 1 receptor (PTH1R) upregulates SLPI expression via the pathways of adenylyl cyclase (AC)/protein kinase A, and Erk. SLPI directly acts in osteoblasts to enhance bone formation by controlling gene expression. Additionally, SLPI promotes the adhesion of osteoblasts to neighboring osteoclasts, thereby increasing direct cell–cell contact. This indirect effect creates a microenvironment in the reversal phase, leading to stimulation of *Slpi* and *Fgf2* expression in osteoblasts, and inhibition of osteoclastic bone resorption.

Bone remodeling is achieved by the orchestration of two cell types that have opposite functions, i.e., bone-resorbing osteoclasts and bone-forming osteoblasts. PTH has been used clinically to stimulate bone formation by enhancing the activities of osteoblasts and osteoclasts in a coordinated manner, although the precise underlying mechanisms remain unclear^11,48^. In this study, we showed that *Slpi* is one of the genes most highly upregulated by PTH in osteoblasts and is critical for the osteoanabolic action of PTH. SLPI exerts two distinct functions: (1) it acts within osteoblasts themselves and enhances bone formation by controlling gene expression, and (2) it enhances adhesive force to neighboring osteoclasts, thereby increasing cell–cell communication between osteoblasts and osteoclasts^2^ (Fig. 5). Therefore, SLPI expression by osteoblasts concurrently regulates the activity of osteoblasts themselves, and their surrounding microenvironment.

Similar bifunctional activity has also been reported for some molecules related to innate immunity and tissue regeneration, such as high mobility group box-1 (HMGB1) and thymidine phosphorylase (TYMP). HMGB1 is a nuclear DNA-binding protein that is involved in transcriptional regulation by p53 or NF-κB. On the other hand, upon its release from dead or damaged cells, HMGB1 also acts as a ligand that activates macrophages and dendritic cells through damage-associated molecular patterns^49^. TYMP, also known as platelet-derived endothelial cell growth factor, catalyzes the hydrolysis of thymidine to ensure the availability of a pyrimidine nucleotide pool^50^. TYMP is secreted and promotes angiogenesis by inducing proliferation and migration of endothelial cells^51,52^. While the functions seem to be independent in these examples, the function of SLPI is characteristic because both functions contribute to the same consequence: an increase in bone mass. SLPI is a reasonable candidate mediator for finely controlling bone metabolism.

In addition to the interaction between osteoblasts and mature osteoclasts, the recruitment of osteoclast precursors (or monocytes/macrophages) might support the anabolic activity of PTH. Indeed, some types of macrophages are associated with PTH anabolism in bone^53^. Previous studies have shown that the migration of osteoclast precursors is regulated by chemokines (e.g., CCR1 chemokines^39^), and growth factors (e.g., platelet derived growth factor BB^40^). In our model, the expression levels of *Ccl5* and *Pdgfa* were increased in *Slpi*-overexpressing osteoblasts, implying that these chemotactic factors may contribute to the attraction of osteoclast precursors (Supplementary Fig. S7a). Moreover, given the partial contribution of SLPI to PTH-induced change in CMI in the present study, there might be an additional, *Slpi*-independent pathway for regulation of bone cell re-distribution. Particularly, cysteine-X-cysteine family chemokine ligand 1 (*Cxcl1*) is highly upregulated in osteoblasts by PTH treatment, and direct osteoclast chemotactic migration^54^. Although the analytical method to evaluate osteoblast-osteoclast precursor communication has not been established yet, the roles of such chemokines in PTH anabolism, as well as migration of osteoclast precursors, should be investigated in the future.

In general, matrix metalloproteases have been considered to play roles in cellular detachment from surrounding components^55^, while serine proteases may also be involved in cell adhesion^56,57^. SLPI plays a role in focal adhesion of cancer cells to the surrounding environment^58^. In our analysis, we focused on the fact that RhoA signaling and ROCK2 protein were affected by the expression of SLPI, as RhoA signaling is associated with cell–cell adhesion through the expression of transmembrane cell adhesion molecules. Given such multiple roles for RhoA signaling, further studies are needed to determine the in vivo role of ROCK proteins in the anabolic state of PTH.

Finally, we should also consider the limitations of this study. Bone histomorphometry showed that intermittent PTH treatment did not significantly change the osteoclast parameters, osteoclast surface/bone surface (Oc.S/BS) or eroded surface (ES/BS), in either WT or *Slpi*-KO mice (Supplementary Table S1). This was partially because bone histomorphometry works well for evaluation of bone formation, but it is less reliable when used to evaluate the dynamism of bone resorption^59^. Oc.S/BS and ES/BS are static measures of resorption surfaces, but provide no direct information concerning the dynamic status of osteoclast activity. In particular, bone histomorphometry did not necessarily reflect PTH-activated bone resorption both in our data and in previous reports by another group^60^. To evaluate the dynamic feature of osteoclast activity influenced by PTH, we analyzed serum CTX levels^61^, and showed that intermittent PTH treatment did not increase bone resorption in WT mice, but did increased it in *Slpi*-KO mice, suggesting a substantial effect of SLPI on bone resorption in PTH anabolism.

In conclusion, based on in vivo screening of gene expression in osteoblasts, we analyzed the bone phenotype of *Slpi*-KO mice and found that SLPI was strongly associated with PTH anabolism. Analysis of the molecular and cellular networks related to PTH anabolism will provide effective information in terms of the optimal usage of this drug and may facilitate the development of therapies for intractable bone diseases.

## Methods

### Mice

C57BL/6J mice were obtained from CLEA Japan (Tokyo, Japan). *Slpi*-KO mice were kindly provided by A. Nakamura’s laboratory (Tohoku Medical and Pharmaceutical University, Sendai, Japan)^23^. The generation of Col2.3-ECFP and TRAP-tdTomato mice was described previously^2,62^. Mice were randomized to the treatment or control group. The mice were bred and maintained under specific pathogen-free conditions at the animal facilities of Osaka University (Osaka, Japan). These mice were maintained in 12-h light/12-h dark cycles, and the housing temperature and humidity were 23 ± 1.5 °C and 45 ± 15%, respectively. We have complied with all relevant ethical regulations for animal testing and research. Animal experiments were performed in accordance with the experimental animal guidelines of Osaka University under approved protocols.

### PTH administration

Intermittent PTH injections were administered to the mice (vehicle, phosphate-buffered saline [PBS], or human PTH (1–34) [teriparatide; Asahi Kasei Pharma Corporation, Tokyo, Japan]). The dose of hPTH (1–34) was 40 µg/kg body weight per day, or vehicle on 5 days per week, delivered via subcutaneous injection for the indicated period, as described previously^18^.

For RNA-seq and qPCR analysis, 12-week-old female Col2.3-ECFP mice were treated with PTH and evaluated at 1 day, 1 week, and 3 weeks later. Osteoblasts were harvested at 24 h after the final injection of intermittent PTH

For continuous PTH administration, osmotic pumps (1004; Alzet, Cupertino, CA, USA) were placed subcutaneously between the shoulder blades. Human PTH (1–34) (40 µg/kg body weight per day) was infused with pumps for the indicated period^63^.

### Isolation of bone cells from adult bones

We isolated bone cells from female adult mice in accordance with a previously reported method^22,64^. Calvaria, humerus, ulnas/radius, hips, tibia, and femur bones were crushed, then washed with Hank’s balanced salt solution. Bone tips were incubated in a 3 mg/mL solution of type I collagenase (Worthington) at 37 °C for 25 min. This incubation in collagenase solution was repeated twice (digestions 1–3). Next, bone pieces were incubated in 5 mM ethylenediaminetetraacetic acid (EDTA) solution (digestion 4). Digestions 1–4 were subjected to flow cytometry for isolation of osteoblasts and MSCs.

### Flow cytometry and cell sorting

Measurements were performed on a FACS Aria II (BD Biosciences, San Jose, CA, USA) and SH800 cell sorter (Sony, Tokyo, Japan), then analyzed using FlowJo software (TreeStar, Ashland, OR, USA). Single-cell suspensions were blocked with anti-CD16/32 antibody (BD Biosciences) for 10 min, followed by staining using flow cytometry (FACS) buffer (1× PBS, 4% heat-inactivated FCS, and 2 mM EDTA) for 15 min. The antibodies for flow cytometry were as follows: anti-CD45-APC (1:50, 103112, BioLegend, San Diego, CA, USA), anti-CD45-FITC (1:50, 103108, BioLegend), anti-CD31-FITC (1:50, 102405, BioLegend), anti-F4/80-PE/Cy7 (1:50, 123113, BioLegend), anti-Ly6C-APC (1:50, 560595, BD Biosciences), anti-Ly6G-BV421 (1:50, 127627, BioLegend), anti-Sca1-APC (1:50, 108111, BioLegend), anti-CD51-PE (1:100, 104105, BioLegend), Streptavidin-PE (1:100, 405203, BioLegend), and Streptavidin-FITC (1:50, 405201, BioLegend), Annexin V-FITC (1:50, 640905, BioLegend), and 7AAD (559525, BD Pharmingen, San Diego, CA, USA).

### RNA-Seq analysis

For in vivo osteoblast analysis, 7AAD^-^Lin^-^CD45^-^ECFP^+^ cells from Col2.3-ECFP mice, treated with intermittent vehicle or PTH (1-34), were isolated using the BD FACSAria II instrument. Osteoblasts were harvested at 24 h after the final injection of PTH or vehicle. Total RNA was purified using an miRNeasy Micro Kit (Qiagen, Hilden, Germany). For in vitro MC3T3-E1 cell analysis, subconfluent cells were digested in QIAZOL (Qiagen) to extract total RNA, in accordance with the manufacturer’s instructions. cDNA was generated using a SMART-Seq v. 4 Ultra Low Input RNA Kit (TaKaRa Clontech, Mountain View, CA, USA). Each cDNA sample was sheared (200–500 bp) using the Covaris S220 system (Covaris, Woburn, MA, USA) and prepared using KAPA Library Preparation Kits (Kapa Biosystems, Wilmington, MA, USA) in accordance with the manufacturer’s instructions. Sequencing was performed on an Illumina HiSeq 2500 platform (Illumina, San Diego, CA, USA) in 75-base single-end mode; Illumina Casava v. 1.8.2 software was used for base-calling. Sequenced reads were mapped to the mouse reference genome sequences (mm10) using TopHat v. 2.0.13 in combination with Bowtie2 v. 2.2.3 and SAMtools v. 0.1.19. The fragments per kilobase of transcript per million mapped reads (FPKM) were calculated using Cuffnorm v. 2.2.1. Bioinformatics analyses were performed using Integrated Differential Expression and Pathway Analysis (iDEP) v. 0.81, and Ingenuity Pathway Analysis software (Ingenuity Systems; Qiagen). The raw data have been deposited in the NCBI Gene Expression Omnibus database (GEO GSE145462).

### Quantitative reverse transcription-polymerase chain reaction

Total RNA and cDNA were prepared using a Maxwell Simply RNA kit (Promega, Madison, WI, USA) and Superscript III reverse transcriptase (Invitrogen, Carlsbad, CA, USA). Real-time PCR was performed with a Thermal Cycler Dice Real-Time System (TaKaRa Bio, Shiga, Japan) using SYBR Premix EX Taq (TaKaRa Bio). The relative levels of the mRNAs of interest were calculated using the 2^−ΔΔCt^ method. The mRNA level of *Gapdh* was used as the internal control. The primer sequences are listed in Supplementary Table S2.

### Microstructure analysis

Micro-computed tomography (CT) was performed on the secondary trabeculae of distal femoral metaphyses. A cone-beam X-ray µCT system (ScanXmate-RB-0-SS150; Comscantecno, Yokohama, Japan) was used to obtain CT images of femoral bone samples. The settings were as follows: tube voltage, 70 kV; tube current, 0.1 mA; and voxel size, 12.0 µm. The 3D images were reconstructed and analyzed using TRI/3D-BON software (Ratoc System Engineering, Kyoto, Japan). Regions of interest were drawn 500 µm from the end of each epiphyseal growth plate to points 1.0 mm along the cortical wall. Bone was segmented from surrounding tissue using a threshold of 700 mgHA/cm^3^.

### Bone histomorphometric analysis

Female mice were injected subcutaneously with 20 mg/kg body weight calcein (Sigma-Aldrich, St. Louis, MO, USA) at 72 and 24 h before sacrifice^65^. Tibias were collected and fixed in 70% ethanol. For histomorphometric analysis, tibias were embedded in glycolmethacrylate and toluidine blue staining was performed on sagittal sections. Calcein labeling was visualized using an inverted light microscope (DM-IL-LED-3; Leica Microsystems, Wetzlar, Germany). Quantitative bone histomorphometric measurements were performed in the secondary spongiosa using a Histometry-RT light microscope (System Supply, Nagano, Japan).

### Measurement of bone metabolic markers

Female mice were fasted for 24 h before blood sampling. Blood was drawn via the inferior vena cava at 24 h after the final injection of PTH or vehicle. Blood specimens were centrifuged at 2000 × *g* for 15 min in gel-separation tubes (TS-701; Sato Kasei Kogyosho Co. Ltd., Tochigi, Japan) to obtain serum. An enzyme-linked immunosorbent assay (ELISA) was used for quantitation of P1NP (Immunodiagnostics Systems, Fountain Hills, AZ, USA) and CTX (RatLaps TM; Immunodiagnostics Systems) in serum.

### Cell culture of osteoblasts

MC3T3-E1 cells were purchased from the American Type Culture Collection (ATCC, Manassas, VA, USA). Primary osteoblasts were isolated from 2-day-old neonatal mouse calvaria as described previously^66^. Briefly, calvaria were subjected to five sequential digestions in 0.25% trypsin, 0.1% EDTA, and 0.32% collagenase type II (Worthington Biochemical Corp., Freehold, NJ, USA) solution for 25 min at 37 °C. Cell fractions 2–5 were collected as primary osteoblasts. Adult primary osteoblasts derived from bone marrow were prepared as described previously^67^. Briefly, bone marrow cells from mouse long bones were seeded (2.5 × 10^5^ cells per cm^2^) and expanded to obtain bone marrow stromal cells (BMSCs). Before differentiation, BMSCs were purified by negative selection with MACS CD45 microbeads (Miltenyi Biotec., Bergisch Gladbach, Germany).

For in vitro differentiation, osteoblasts were cultured with 50 µg/mL ascorbic acid (Wako Pure Chemical Co, Tokyo, Japan) and 10 mM β-glycerophosphate (Sigma-Aldrich) in α-minimum essential medium (MEM) supplemented with 10% (v/v) fetal bovine serum, 0.22% sodium bicarbonate, 100 U/mL penicillin, and 100 µg/mL streptomycin.

ALP staining was performed using naphthol-ASMX as the substrate and Fast Blue RR salt as the coupler. ALP activity was detected using pNPP (4-nitrophenyl phosphate) with a TRACP & ALP Assay Kit (TaKaRa) as a colorimetric substrate, then measured by spectrophotometry at 405 nm. Staining and quantification of ALP activity were performed at 3 and 7 days after differentiation of MC3T3-E1 cells and primary osteoblasts, respectively.

Alizarin Red S staining and its quantification were performed using a Mineralization Quantification Kit (PG-Research, Tokyo, Japan). Staining was performed at 14 days after differentiation of MC3T3-E1 osteoblasts and primary osteoblasts. Quantification of Alizarin Red S staining was via extraction with formic acid. The amount of dye released was quantified by spectrophotometry at 405 nm.

For the generation of conditioned medium, supernatants from subconfluent cultures of MC3T3-E1 cells were collected and passed through a 0.45 µm filter to eliminate cells, diluted with one volume of α-MEM, and used as conditioned medium.

For analysis of PTH1R signaling, osteoblasts were cultured under serum-free conditions for 12 h and topically treated with the following stimulators or inhibitors at the indicated concentrations:

Human PTH (1-34): PBS; 24.3 nM

Forskolin (Sigma-Aldrich): DMSO; 5 µM

PMA (Sigma-Aldrich): DMSO; 100 nM

A23187 (Sigma-Aldrich): DMSO; 5 µM

Rp-cAMPS (Sigma-Aldrich): DMSO; 25 µM

H89 (Calbiochem; Sigma-Aldrich): DMSO; 1 µM

U0126 (Cayman Chemical, Ann Arbor, MI, USA): DMSO; 10 µM

### Retroviral and lentiviral gene transfer

The retroviral vector, pMX-*Slpi*-IRES-Puro, was constructed by inserting *Eco*RI and *Xho*I fragments of the full-length mouse *Slpi* cDNA into pMX-IRES-Puro. Retroviral packaging was performed by transfecting the plasmids into Plat-E (Cell Biolabs, Inc., CA, USA) cells using FuGENE6 (Promega)^68^. Two days after inoculation, MC3T3-E1 cells or primary osteoblasts were incubated with viral supernatant from pMX-IRES-Puro and pMX-*Slpi*-IRES-Puro virus-producing Plat-E cells, together with polybrene (8 µg/mL) for 12 h. Seven days after infection, the cells were treated with puromycin (10 µg/mL) to select stable transformants.

To generate the MC3T3-E1-EGFP cell line, enhanced green fluorescent protein (EGFP) (pEGFP-C1; Clontech) was inserted into the CSII-EF-MCS vector (provided by Dr. Miyoshi of RIKEN-BRC, Tsukuba, Japan) and transfected into HEK293T cells (ATCC) with packaging plasmids^69^. Two days after inoculation, MC3T3-E1 cells were incubated with lentiviral supernatant from virus-producing HEK293T cells, together with polybrene (8 µg/mL), for 12 h. Stable transformants were selected using an SH800 cell sorter (Sony).

### Evaluation of osteoblast proliferation

The proliferation of osteoblasts was assessed by counting cultured osteoblasts using a TC10 Automated Cell Counter (Bio-Rad, Hercules, CA, USA). Briefly, osteoblasts were seeded into six-well plates at 1 × 10^4^ /cm^2^. After 3 and 5 days, cells were suspended by incubation with 0.25% trypsin-EDTA (Sigma-Aldrich), and live cells were enumerated by trypan blue staining (Sigma-Aldrich).

### Culture of primary osteoclasts

Nonadherent bone marrow cells derived from mice were seeded (1.5–2.0× in a 10 cm Petri dish) and cultured in the presence of 30 ng/mL M-CSF (R&D Systems, Minneapolis, MN, USA). After 3 days, adherent cells were collected in enzyme-free cell dissociation buffer (Millipore, Burlington, MA. USA) at 37 °C and used as bone marrow macrophages (BMMs). BMMs were further cultured in the presence of 50 ng/mL soluble RANKL (Oriental Yeast Co., Ld., Tokyo, Japan) and 10 ng/mL M-CSF to generate osteoclasts^70^. Three days later, TRAP staining was conducted using an Acid Phosphatase, Leukocyte (TRAP) Kit (Sigma-Aldrich). TRAP^+^ multinucleated (>3 nuclei) cells were regarded as mature osteoclasts.

To evaluate bone resorptive activity, osteoclasts were cultured on RepCell dishes for 5 days. Next, the cells were transferred to an Osteo Assay Surface (Corning, Corning, NY, USA). After 48 h, resorption pits were observed under an inverted light microscope (DM-IL-LED-3; Leica Microsystems), and the binarized areas of resorption pits were measured using NIS Elements integrated software (Nikon, Tokyo, Japan).

### Mass spectrometry

Protein samples in buffer solution were precipitated by methanol-chloroform addition and then dissolved in RapiGest (Waters, Milford, MA, USA). The samples were reduced with 10 mM dithiothreitol followed by alkylation with iodoacetamide, then digested by trypsin and purified using a C18 tip (GL Science, Tokyo, Japan). Trypsin-digested peptides were subjected to liquid chromatography-tandem mass spectrometry, using an Aurora column (Ionoptics) on a Nano Elute nanoLC system coupled to a trapped ion mobility spectrometry-time-of-flight mass spectrometer (Bruker, Billerica, MA, USA). The column temperature was set to 50.0 °C. The mobile phase consisted of water with 0.1% formic acid (solvent A) and acetonitrile containing 0.1% formic acid (solvent B). Peptides were eluted at a gradient of 4–30% B for 20 min with a flow rate of 400 nL/min. The mass-scanning range was set from 100 to 1700 *m/z* and a custom ion mobility revolution mode was used (range: 0.6–1.50 Vs/cm^2^). The ion spray voltage was set at 1.6 kV in the positive-ion mode. The MS/MS spectra were acquired by automatic switching between MS and MS/MS modes. Data Analysis software (Bruker) was used to process the mass data. Peptides were identified by database searching using our in-house MASCOT Server (Matrix Science, London, UK). The precursor and fragment-searching mass tolerances were set to 20 ppm and 0.05 Da, respectively. Carbamidomethylation of cysteine was set as a static modification, while the oxidation of methionine, acetylation of protein N-term, and deamination of N,Q were set as variable modifications. The MASCOT Server search results were analyzed by Scaffold (Proteome Software Inc., Portland, OR, USA) and the results were limited to those with a GO annotation of “osteoblast.” Finally, the quantitative values (normalized total spectra) of mock and immunoprecipitation samples were compared.

### Immunoblotting analysis

Cells were lysed in RIPA buffer supplemented with protease inhibitor cocktail (Sigma-Aldrich). Conditioned media were mixed with four volumes of chilled acetone. Proteins were precipitated overnight at −30 °C and dissolved in RIPA buffer. Protein concentrations were measured by bicinchoninic acid protein assay (Thermo Scientific Inc., Waltham, MA, USA). Cell lysates were boiled in sodium dodecyl sulfate loading dye. Equal amounts of total protein were separated on 4–20% mini-Protean TGX gradient gels (Bio-Rad) and transferred to polyvinylidene difluoride membranes (Immobilon P). The membranes were incubated with the primary and secondary antibodies. Finally, bands were visualized with the electrochemiluminescence reagents (GE Healthcare, Chicago, IL, USA) and using an ImageQuant LAS-4000 mini system (GE Healthcare). The antibodies for immunoblotting were as follows: anti-β-catenin (1:1000, 610153, BD Biosciences), anti-SLPI (1:1000, AF1735, R&D Systems), anti-ROCK1 (1:1000, ab134181, Abcam, Cambridge, UK), anti-ROCK2 (1:1000, ab125025, Abcam) and anti-β-actin-HRP (1:5000, ab20272, Abcam), goat anti-mouse IgG-HRP (1:1000, sc-2005, Santa Cruz Biotechnology, Santa Cruz, CA, USA), rabbit anti-goat IgG-HRP (1:1000, sc-2768, Santa Cruz Biotechnology).

### Coculture of osteoblasts and osteoclasts

For time-lapse imaging, pMX-IRES-Puro or pMX-*Slpi*-IRES-Puro were introduced into MC3T3-E1-EGFP cells. Stable transformants were selected with puromycin (10 µg/mL) for 3 days. Subsequently, the transformants were cocultured with primary osteoclasts. MC3T3-E1-EGFP cells and TRAP-tdTomato^+^ osteoclasts were cocultured on a collagen gel-coated glass-bottom dish. MC3T3-E1 cells were cultured at a density of 750 cells/cm^2^, while mature osteoclasts were cultured at a 1:3 ratio of MC3T3-E1 cells to mature osteoclasts. To assess the effect of SLPI antibody on cell-cell contact, SLPI antibodies (AF1274, R&D Systems; S0635, US Biological Life Sciences, Salem, MA, USA) 12.5 µg/mL, or control goat IgG (Santa Cruz Biotechnology) were added before time-lapse imaging. After 3 days of coculture, time-lapse images were acquired using an A1 confocal microscope (Nikon). To assess the effect of Y27632 on cell-cell contact, MC3T3-E1 cells were exposed to 10 µM Y27632 for 12 h. The reagent was removed before coculture to prevent it from acting on osteoclasts. For tracking of cell-cell contact, images were first automatically binarized by Otsu’s thresholding method using NIS Elements integrated software (Nikon). The surface tool of Imaris software (Bitplane, Zürich, Switzerland) was used to track cell movement. Only EGFP-stained surface objects ≥100-µm^2^ and tdTomato-stained surface objects ≥1000-µm^2^ were analyzed, to exclude extracellular vesicles from osteoblasts and tdTomato^+^ mononuclear cells. A new channel representing the colocalized area of EGFP and tdTomato voxels was created automatically by the surface tool. Finally, the contact duration was determined by tracking this new channel using Imaris software.

To assess the effects of cell-cell interaction on the differentiation and activity of each cell type, osteoblasts (or MC3T3-E1 cells) and primary osteoclasts were first cultured separately. For osteogenic differentiation, primary osteoblasts and primary osteoclasts were each seeded at a density of 1.5 × 10^4^ cells/cm^2^. Osteoblasts were differentiated for 14 days in the presence of RANKL and M-CSF. Osteoclasts were added at 3-day intervals during osteoblast differentiation. For osteoclast differentiation, osteoblasts (or MC3T3-E1 cells) and BMMs were seeded at densities of 1.5 × 10^4^/cm^2^, and 3.75 × 10^4^/cm^2^ respectively. Then, osteoclast differentiation was induced in the presence of RANKL and M-CSF for 5 days as described above. For the resorption pit assay, BMMs were differentiated into mature osteoclasts on 6 cm^2^ RepCell dishes for 5 days. Then osteoclasts were cocultured with MC3T3-E1 cells on an Osteo Assay Surface (Corning) for 48 h in the presence of RANKL and M-CSF to evaluate resorption activity.

To evaluate gene expression in coculture, MC3T3-E1-EGFP cells were differentiated for 14 days. BMMs derived from TRAP-tdTomato^+^ mice were differentiated into mature osteoclasts on 6-cm^2^ RepCell dishes for 5 days. For juxtacrine interactions, MC3T3-E1 cells and osteoclasts were mixed at a ratio of 2:1 and cocultured for 24 h at a density of 3.75 × 10^4^ cells/cm^2^. For paracrine interactions, osteoclasts were cocultured for 24 h, using a Millicell Hanging Cell culture insert with a 1-µm pore PET membrane (Millipore), with MC3T3-E1 cells at a density of 2.5 × 10^4^ cells/cm^2^. Inserts lacking cells were used as controls. Cells were collected using collagenase type II, and the EGFP^+^ tdTomato^-^ (MC3T3-E1 cells) and EGFP^-^tdTomato^+^ (primary osteoclasts) fractions were sorted using an SH800 cell sorter (Sony).

### Intravital multiphoton bone imaging

Calvarial bone tissues of female Col2.3-ECFP/TRAP-tdTomato/*Slpi*^wt/wt^ and Col2.3-ECFP/TRAP-tdTomato/*Slpi*^KO/KO^ mice (12–20 weeks of age) were used for intravital bone imaging^2^. In this experiment, parietal lamina externa and intervening spongy bone (diploe) were observed. For surgery and continuous intravital imaging, mice were anesthetized with isoflurane (Escain; 2.5% vaporized in an 80:20 mixture of O_2_ and air). The frontal and parietal bones were exposed and immobilized in a custom-made stereotactic holder.

The imaging system consisted of an upright multiphoton microscope (A1R MP+; Nikon) with a 25× water-immersion objective (CFI75 Apo 25XC W 1300; Nikon). The system was driven by lasers (Chameleon Vision II Ti: Sapphire; Coherent, Inc, Santa Clara, CA, USA); the main laser was tuned to 860 nm to detect ECFP and second harmonic generation (SHG), and the sub-laser was tuned to 1040 nm to detect tdTomato. Using a Nikon upright microscope, multi-fluorescence images were acquired directly using four external non-descanned detectors equipped with dichroic and emission filters including an infrared-cut filter (DM685), three dichroic mirrors (DM458, DM506, and DM605), and three emission filters (417/60 for the SHG image, 480/40 for ECFP, 583/22 for tdTomato).

### Cell mixture analysis

Cell mixtures were evaluated using the CMI^2^. To obtain the tiling images, snapshot image stacks of 16 continuous visual fields were collected across the sagittal suture at depths of 50–200 μm below the skull bone surface (5-μm vertical steps) using 1.0× zoom and 512 × 512 X–Y resolution. Maximum-intensity projection (MIP) images of tdTomato^+^ mature osteoclasts and ECFP^+^ mature osteoblasts were stitched to produce a tiling image. The threshold for binarization was determined automatically using Otsu’s method for a tiling image with gamma correction. Next, the MIP images were divided to create 16 images; only images in which the ratio of red area to cyan area ranged between 0.5 and 2 were analyzed. CMI indicates the degree of mixing of two cell types in the range from 0 to 1, and was calculated by hierarchical clustering. Euclidean distances were calculated between each pixel in the binarized cell area, and Ward’s method was used for the hierarchical clustering algorithm. Each pixel was initially considered a cluster of its own. Small clusters were combined sequentially into larger clusters, until all pixels converged into one cluster regardless of the color of individual pixels. The impurity of the clusters was calculated using a Gini-like formula, and CMI was defined as the area under the curve. Cell mixture analysis was performed using R software (ver. 3.6.0; R Development Core Team, Vienna, Austria) and the EBImage package (ver. 4.20.0).

### Three-dimensional colocalization analysis

The numbers of osteoblast–osteoclast contacts were determined using Imaris. A Sobel filter was used to detect cell edges, in all depth slices and channels (3 × 3; four directions for images acquired by the Nikon upright microscope with a 1.0× zoom). We next adjusted the raw images to yield edge-enhanced images. The surface tool in Imaris was used to perform automatic cell-surface segmentation of each cyan-positive and tdTomato-positive cell evident on intravital time-lapse bone imaging. Cyan-stained surface objects ≤125 µm^3^ in volume, and tdTomato-stained surface objects ≤1000 µm^3^ in volume, were not included in the analysis, as these objects were unlikely to represent cells. The surface tool was then used to detect osteoblast–osteoclast contacts, and to automatically create a new channel (yellow) for colocalized cyan and tdTomato voxels.

### Histological analysis

Mice were perfused with 4% paraformaldehyde (PFA) for fixation and dissected femoral bones were further fixed with 4% PFA and 20% sucrose for 4 h at 4 °C. Samples were embedded in Super Cryoembedding medium (Section-LAB Co. Ltd) and frozen in chilled hexane (Wako) using dry ice^2^. Frozen samples were cut into 10 µm sections with a cryostat (Leica, CM3050). Images were acquired with an A1 confocal microscope (Nikon). Specific parameters (excitation laser wavelength, dichroic mirrors, and emission filters, respectively) were used for detecting fluorescence of ECFP (457 nm, DM515, and BP482/35) and tdTomato (561 nm, DM640, and BP595/50) (Nikon).

### Statistical analysis

Statistical analysis was performed using unpaired two-tailed *t*-tests for comparisons between two groups, and using analysis of variance (ANOVA) with the Dunnett’s, Tukey’s, and Šidák’s post hoc test for comparisons among three or more groups. Welch’s ANOVA followed by Bonferroni’s multiple comparisons test was used when the data demonstrated unequal variance. Statistical significance was recorded when *p* < 0.05. Biological replicates comprised samples from different mice. The results are representative of more than three independent experiments. The minimum possible numbers of samples were used. Mice were excluded if they exhibited any abnormalities in size, weight, or apparent symptoms of disease, before performing the experiments; however, no such phenomena were observed.

### Reporting summary

Further information on research design is available in the Nature Research Reporting Summary linked to this article.

## Supplementary information

Supplementary Information

Supplementary Movie 1

Supplementary Movie 2

Supplementary Movie 3

Supplementary Movie 4

Supplementary Movie 5

Supplementary Movie 6

Reporting Summary

Description of Additional Supplementary Files

## Data Availability

The datasets are available from the corresponding authors on reasonable request. Raw RNA-Seq data is available from the Gene Expression Omnibus (accession number and hyperlinks: GSE145462).
