# Supplementary material for: SLPI is a critical mediator that controls PTH-induced bone formation
Source: Nat Commun. 2021 Apr 9;12:2136. doi: 10.1038/s41467-021-22402-x (PMC8035405; doi:10.1038/s41467-021-22402-x)
Supplement: Supplementary file 1 — Supplementary Information [file 41467_2021_22402_MOESM1_ESM.pdf]

Supplementary information

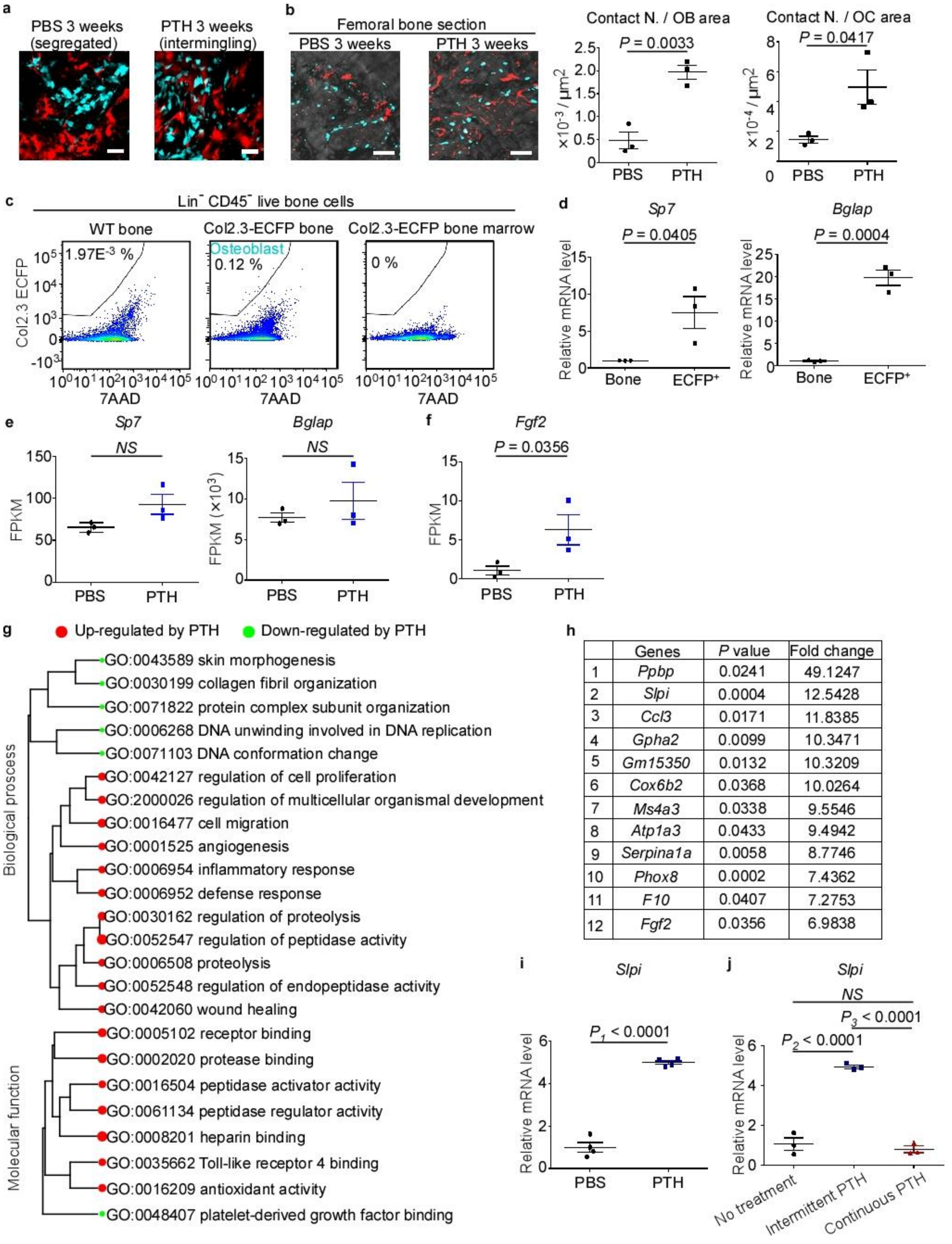

	Genes	*P* value	Fold change
1	*Ppbp*	0.0241	49.1247
2	*Slpi*	0.0004	12.5428
3	*Ccl3*	0.0171	11.8385
4	*Gpha2*	0.0099	10.3471
5	*Gm15350*	0.0132	10.3209
6	*Cox6b2*	0.0368	10.0264
7	*Ms4a3*	0.0338	9.5546
8	*Atp1a3*	0.0433	9.4942
9	*Serpina1a*	0.0058	8.7746
10	*Phox8*	0.0002	7.4362
11	*F10*	0.0407	7.2753
12	*Fgf2*	0.0356	6.9838

Supplementary Figure 1. RNA-Seq-based transcriptional profiling of osteoblasts after PTH treatment.

a, MIP images of skull bone tissues from Col2.3-ECFP/TRAP-tdTomato mice

under control conditions (left) and after intermittent PTH treatment for 3 weeks (right). Images were acquired by intravital multi-photon microscopy (Representative image, n = 3 biologically independent experiments). Cyan, mature osteoblasts expressing Col2.3-ECFP; red, mature osteoclasts expressing TRAP-tdTomato. Scale bar, 50 μm. **b,** Left panel shows histological analysis of a femoral metaphyseal region of Col2.3-ECFP/TRAP-tdTomato mice (Representative images, n = 3 mice per group). Cyan, mature osteoblasts; red, mature osteoclasts. Scale bar, 100 μm. Right panel shows numbers of contacts between osteoblasts and osteoclasts; data were normalized to the $ECFP^+$ osteoblasts (OB) area, and $tdTomato^+$ osteoclasts (OC) area, respectively (n = 3 mice per group). **c,** FACS separation of $ECFP^+$ cells from the indicated tissues. BM, bone marrow. **d,** Gene expression profile of $ECFP^+$ cells (quantitative reverse transcription-polymerase chain reaction, qRT-PCR) (n = 3 biologically independent samples per group). **e,** Expression levels of osteoblast marker genes in $ECFP^+$ cells harvested from mice treated intermittently with phosphate-buffered saline (PBS) or PTH (RNA-Seq) (n = 3 biologically independent samples per group). FPKM, fragments per kilobase of exon per million reads mapped. **f,** Expression level of *Fgf2* in $ECFP^+$ cells from mice treated intermittently with PBS or PTH (RNA-Seq) (n = 3 biologically independent samples per group). FPKM, fragments per kilobase of exon per million reads mapped. **g,** Enriched Gene Ontology (GO) categories of differentially expressed genes between control and intermittently PTH-treated osteoblasts (RNA-Seq). Gene sets closer on the tree share more genes. Dot size is proportional to adjusted p-value. **h,** Top 12 upregulated genes in $ECFP^+$ cells induced by PTH treatment (n = 3 biologically independent samples per group). **i,** Validation that the *Slpi* gene is regulated by PTH (qRT-PCR) (n = 4 biologically independent samples per group). **j,** *Slpi* expression in control (untreated) $ECFP^+$ cells or cells subjected to intermittent and continuous PTH treatment for 3 weeks (qRT-PCR) (n = 3 biologically independent samples per group). Data are means ± SEM. NS, not significant. Statistical significance was determined by two-tailed Student's t-test (**b**, **d**, **e**, **f**, **h**, **i**), and ANOVA with Tukey's test (**j**). (**i**) P_1 exact value = 3.3E^{-6}. (**j**) P_2 exact value = 3.2E^{-5}, P_3 exact value = 2.2E^{-5}.

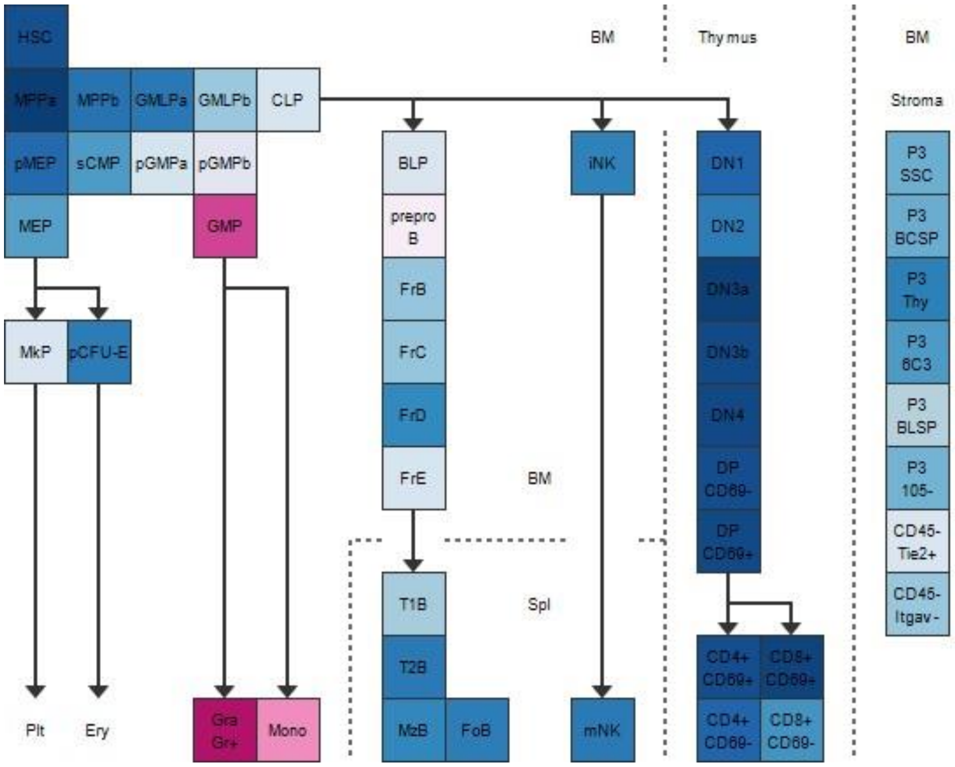

Supplementary Figure 2. Expression of SLPI in bone marrow.

Relative expression levels of *Slpi* in hematopoietic and stromal populations in bone marrow tissue, as determined by microarray analysis. Data are accessible through the Gene Expression Commons platform (https://gexc.riken.jp).

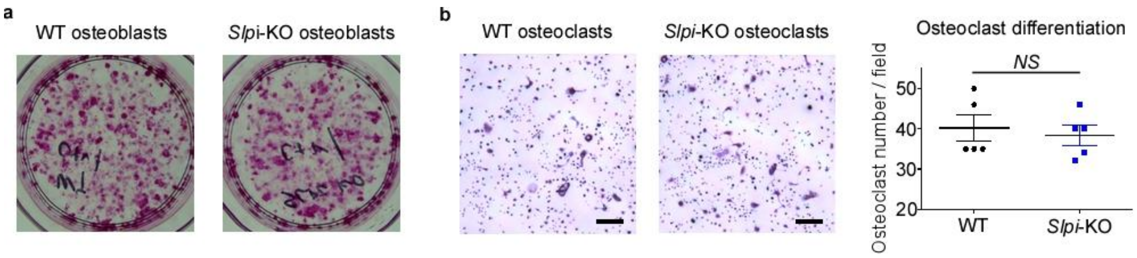

Supplementary Figure 3. Bone phenotype of *Slpi*-KO mice.

a, Alizarin Red S staining of primary osteoblasts from WT and *Slpi*-KO mice. Osteoblasts were incubated in osteogenic medium for 14 days. β-glycerophosphate was used at a concentration of 10 mM. **b,** TRAP staining of primary osteoclasts from WT and *Slpi*-KO mice. Scale bar, 200 μm. Right, numbers of TRAP$^+$ cells (n = 5 biological replicates per group). Data are means ± SEM. NS, not significant. Statistical significance was determined by two-tailed Student's t-test (**b**).

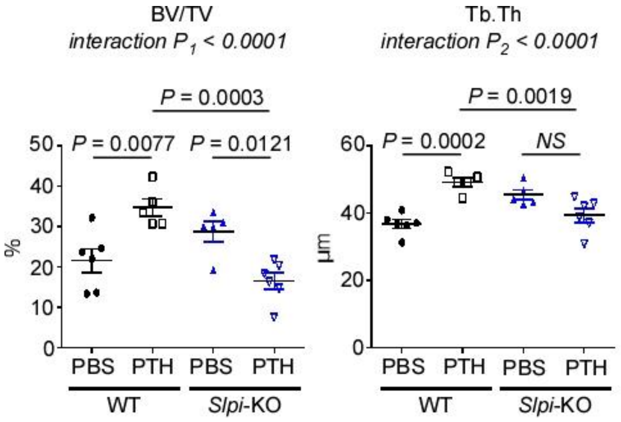

Supplementary Figure 4. Microcomputed tomography (µCT) analysis of male *Slpi*-KO mice.

Ratio of bone volume to total bone volume (BV/TV), and trabecular thickness (Tb.Th) values obtained from the secondary trabeculae of distal metaphyses in WT and *Slpi*-KO male mice treated with vehicle or intermittent PTH (WT PBS: n = 6 mice, WT PTH: n = 5 mice, *Slpi*-KO PBS: n = 5 mice, *Slpi*-KO PTH: n = 6 mice). Data are means ± SEM. NS, not significant. Statistical significance was determined by ANOVA with Tukey's test. P_1 exact value = 7.2E^{-5}, P_2 exact value = 1.6E^{-5}.

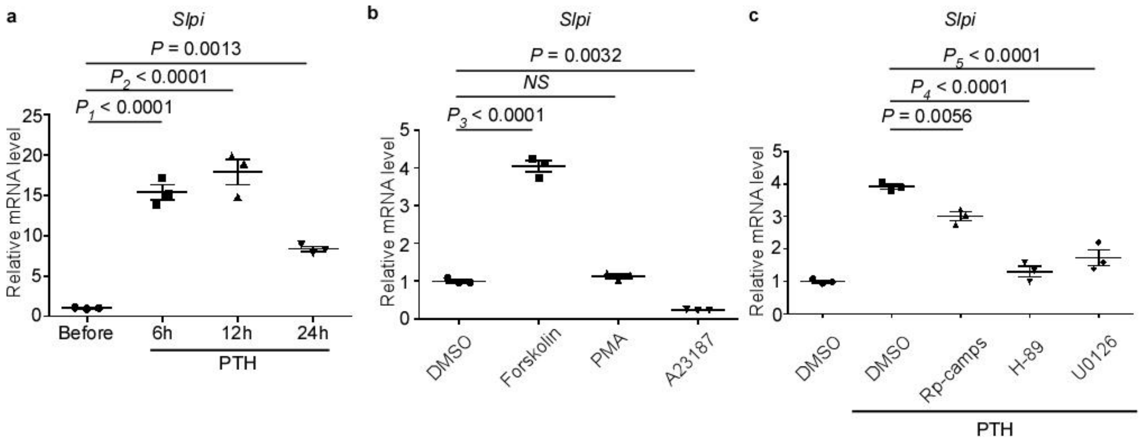

Supplementary Figure 5. PTH regulates *Slpi* expression via protein kinase A (PKA) and Erk signaling.

a, mRNA levels of *Slpi* in MC3T3-E1 cells. MC3T3-E1 cells were differentiated in osteogenic medium for 14 days and starved of serum for 12 h. The cells were stimulated with 24.3 nM PTH, and *Slpi* expression levels were evaluated before and at 6, 12, and 24 h after PTH treatment (n = 3 cells per group). **b,** *Slpi* mRNA levels in MC3T3-E1 cells. MC3T3-E1 cells were differentiated in osteogenic medium for 14 days and starved of serum for 12 h. The cells were treated with the indicated stimulators. After 6 h, the levels of *Slpi* mRNA were measured (qRT-PCR) (n = 3 cells per group). **c,** *Slpi* mRNA levels in MC3T3-E1 cells. MC3T3-E1 cells were differentiated in osteogenic medium for 14 days and starved of serum for 12 h. The cells were pretreated with H89 and Rp-cAMPS for 30 min and with U0126 for 12 h. Next, the cells were stimulated with human PTH. The levels of *Slpi* mRNA were measured after 6 h (qRT-PCR) (n = 3 cells per group). Data are means ± SEM. NS, not significant. Statistical significance was determined by ANOVA with Dunett's test (**a**, **b**, **c**). (**a**) P_1 approximate value < E^{-15}, P_2 approximate value < E^{-15}. (**b**) P_3 approximate value < E^{-15}. (**c**) P_4 approximate value < E^{-15}, P_5 approximate value < E^{-15}.

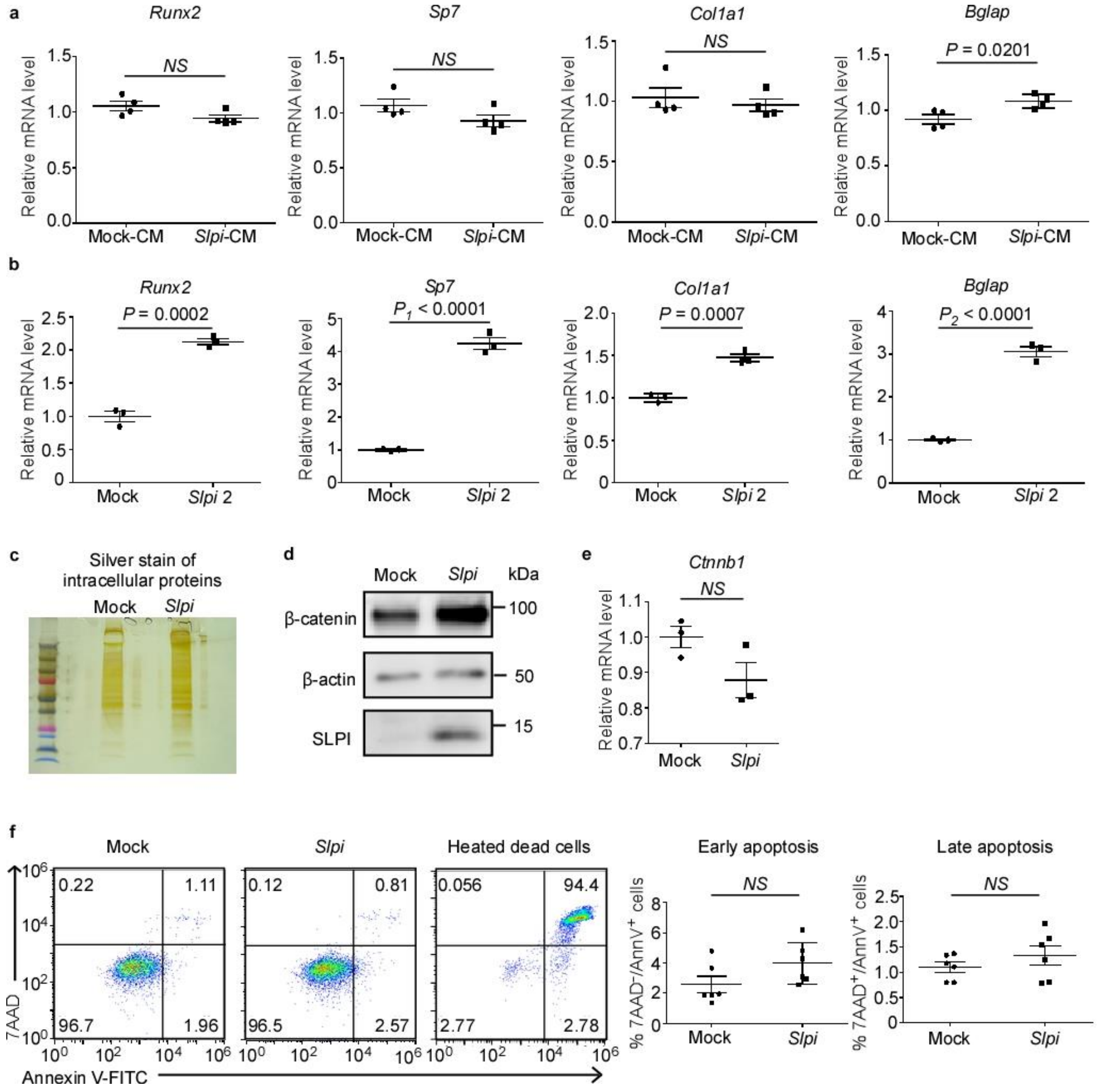

Supplementary Figure 6. Direct effect of *Slpi* on osteoblasts.

a, qPCR analysis of MC3T3-E1 cells treated with *Slpi* conditioned medium under osteogenic conditions for 3 days (n = 3 biological replicates per group). For the generation of conditioned medium, the supernatants from subconfluent cultures of MC3T3-E1 cells were collected and passed through a 0.45-μm filter to eliminate cells. Supernatants were then diluted with one volume of α-MEM, and used as the conditioned medium. **b,** qPCR analysis of MC3T3-E1 cells transfected with pMX-*Slpi*-puro or pMX-puro (n = 3 biological replicates per group). Cells were cultured in osteogenic differentiation medium for 3 days. Subsequently, the expression levels of osteoblast transcript regulators (*Runx2*, *Sp7*) and differentiation marker genes (*Bglap*, Col1a1) were quantified. **c,** Silver staining of intracellular proteins from control or *Slpi*-overexpressing MC3T3-E1

cells. Total protein levels were determined by bicinchoninic acid (BCA) assay. **d,** β-catenin levels in MC3T3-E1 cells (Representative blot, n = 3 biologically independent experiments). Control (mock) and *Slpi*-overexpressing (*Slpi*) MC3T3-E1 cells were seeded, incubated until 60% confluence, lysed, and subjected to immunoblotting analysis. **e,** mRNA levels of β-catenin in control and *Slpi*-overexpressing MC3T3-E1 cells (n = 3 biological replicates per group). **f,** Control (mock) or *Slpi*-overexpressing MC3T3-E1 cells were examined by flow cytometry with co-staining of annexin V and 7-AAD. Heated dead cells were used as the positive control. Percentages of cells are shown. Right, percentages of apoptotic cells (n = 6 biological replicates per group). Data are means ± SEM. NS, not significant. Statistical significance was determined by two-tailed Student's t-test (**a**, **b**, **e**, **f**). (**b**) P_1 exact value = 5.7E^{-5}, P_2 exact value = 6.4E^{-5}.

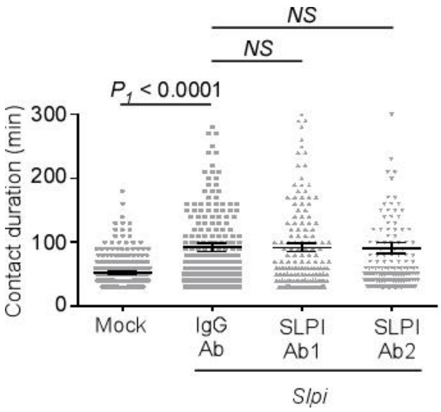

Supplementary Figure 7. Effects of SLPI antibodies on colocalization of osteoblasts and osteoclasts induced by SLPI.

Mock or *Slpi*-overexpressing MC3T3-E1-EGFP cells were cocultured with tdTomato^{+} primary osteoclasts (Mock: n= 267 cells examined over 6 independent experiments, *Slpi* IgG Ab: n = 274 cells examined over 6 independent experiments, *Slpi* SLPI Ab1: n = 167 cells examined over 5 independent experiments, *Slpi* SLPI Ab2: n = 158 cells examined over 5 independent experiments). The contact duration was analyzed in the absence or presence of two commercially available SLPI neutralizing antibodies. Data are means ± SEM. NS, not significant. Statistical significance was determined by ANOVA with Tukey's test. P_1 exact value = 2.4E^{-7}.

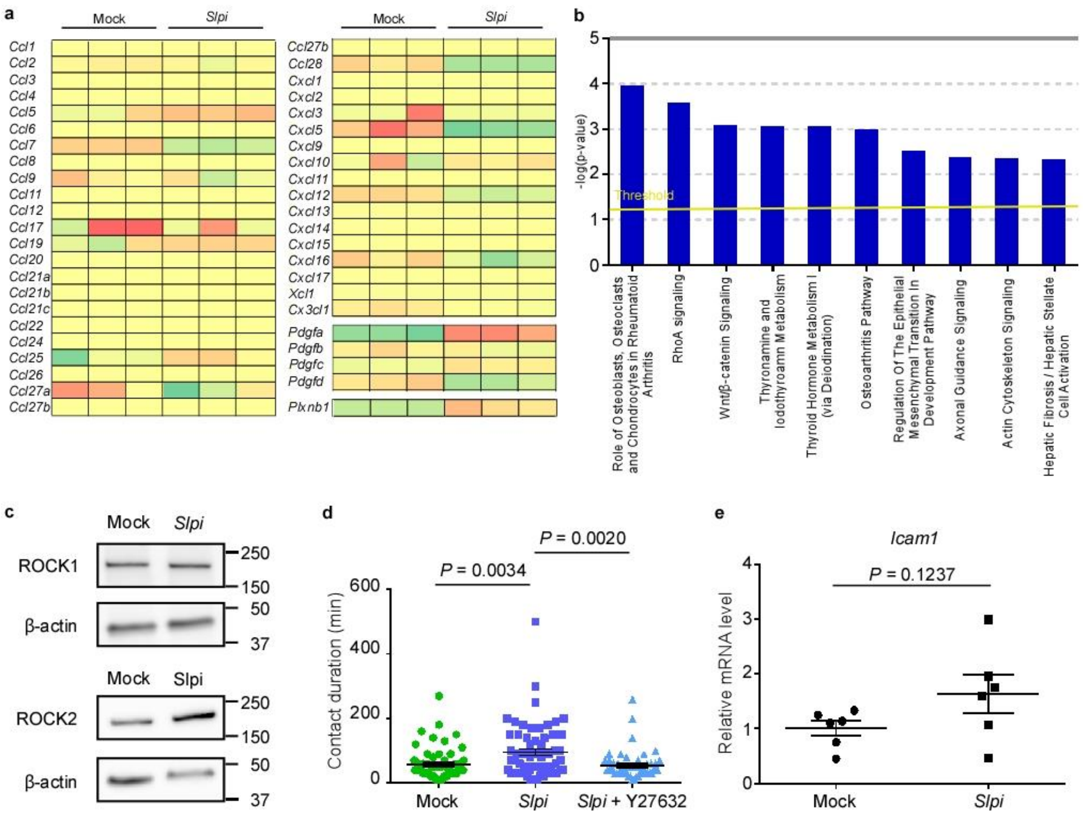

Supplementary Figure 8. Mechanistic insight of osteoblast-osteoclast colocalization via SLPI.

a, RNAseq data representing chemokines, growth factors, and an axon guidance molecule: green, downregulation; red, upregulation (n = 3 biological replicates per group). **b,** List of canonical pathways affected by SLPI based on RNA-sequencing of mock and *Slpi*-overexpressing MC3T3-E1 cells (n = 3 biological replicates per group). Top 10 categories are shown. Data were calculated using Ingenuity Pathway Analysis (IPA). Genes associated with RhoA signaling, and Wnt/β-catenin signaling were highly-ranked in the *Slpi*-overexpressing cells. **c**, Immunoblotting analysis of ROCK proteins in MC3T3-E1 cells (Representative blot, n = 2 biologically independent experiments). **d**, Effects of ROCK inhibitor Y27632 on the colocalization of osteoblasts and osteoclasts (Mock: n= 56 cells examined over 5 independent experiments, *Slpi*: n = 64 cells examined over 5 independent experiments, *Slpi* + Y27632: n = 50 cells examined over 5 independent experiments). Mock or *Slpi*-overexpressing MC3T3-E1-EGFP cells were cocultured with tdTomato$^+$ primary osteoclasts. **e**, *Icam1* mRNA expression levels in MC3T3-E1 cells (n = 6 biological replicates per group). Data are means ± SEM. Statistical significance was determined by ANOVA with Tukey's test (**d**), and two-tailed Student's t-test (**e**).

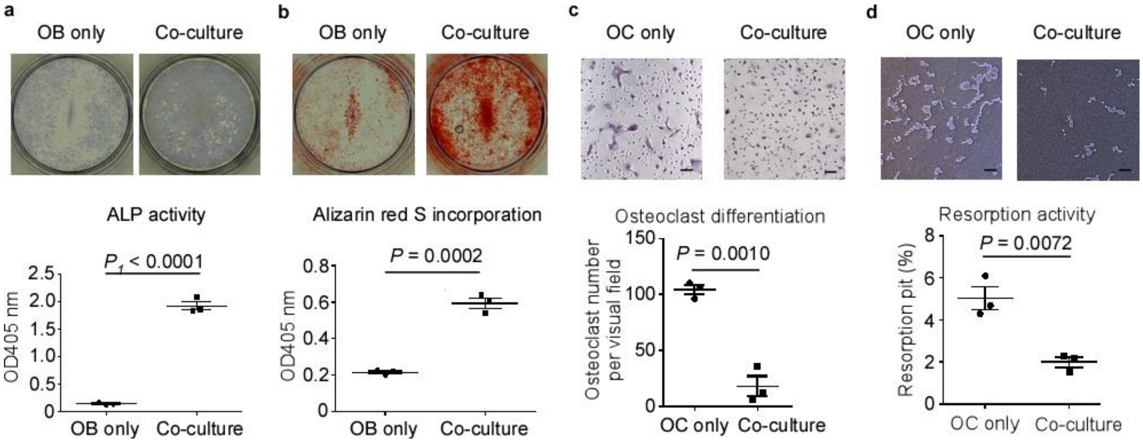

Supplementary Figure 9. Consequences of osteoblast and osteoclast colocalization.

a, b, Representative images of adult primary bone marrow osteoblasts in the presence or absence of primary osteoclasts, assessed by alkaline phosphatase (ALP) staining (**a**) and Alizarin Red S staining (**b**). β-glycerophosphate was used at a concentration of 10 mM. After 14 days of differentiation, quantification of ALP activity and Alizarin Red S was measured (n = 3 biological replicates). **c,** Representative images of primary osteoclasts in the presence or absence of MC3T3-E1 cells, assessed by TRAP staining. $TRAP^+$ multinuclear cells were considered mature osteoclasts (n = 3 biological replicates). Scale bar, 100 µm. **d,** Effects of the presence of MC3T3-E1 cells on the generation of bone resorption pits. Mature osteoclasts were transferred to a Corning Osteoassay Surface in the presence or absence of differentiated MC3T3-E1 cells. After 48 h, resorption pits were visualized. Scale bar, 100 µm. Lower panel, area of resorption pits (%) (n = 3 biological replicates per group). Data are means ± SEM. NS, not significant. Statistical significance was determined by two-tailed Student's t-test (**a**, **b**, **c**, **d**). (**a**) P_1 exact value = $2.1E^{-5}$.

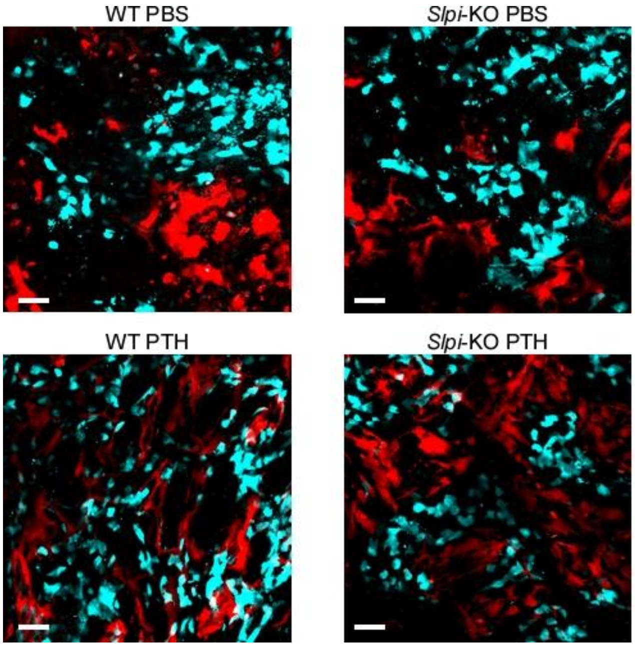

Supplementary Figure 10. Magnified view of tiling images.

Magnified view of tiling maximum-intensity projection (MIP) images of skull tissues obtained by intravital microscopy, as shown in **Fig. 4a**. Left panel, tiling image of Col2.3-ECFP/TRAP-tdTomato/$Slpi^{WT/WT}$ mice; right panel, image of Col2.3-ECFP/TRAP-tdTomato/$Slpi^{KO/KO}$ mice at 3 weeks after intermittent PBS (upper panel) or PTH (lower panel) treatment (Representative image, n = 6 biologically independent experiments). Cyan, mature osteoblasts; red, mature osteoclasts. Scale bars, 50 μm.

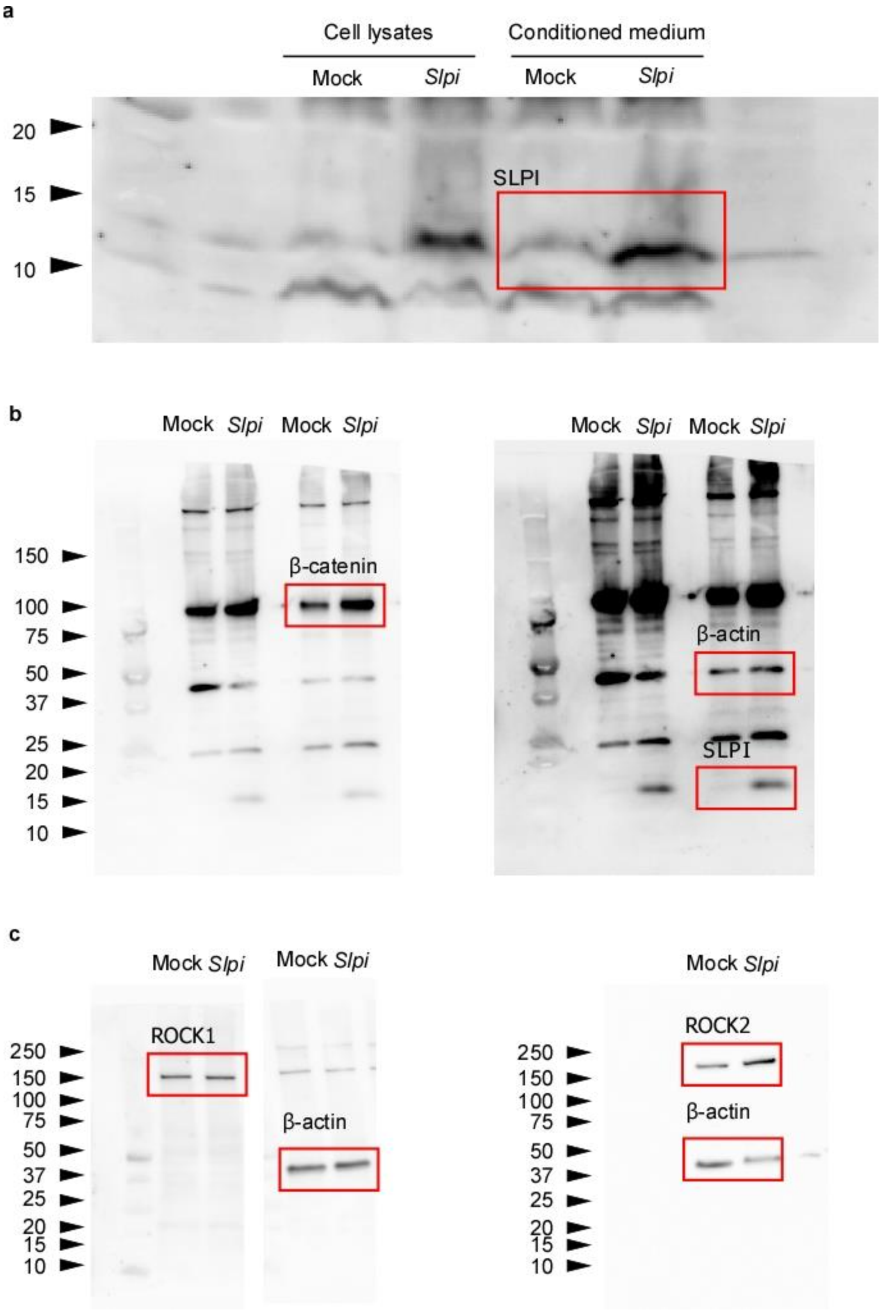

Supplementary Figure 11. Source figures for immunoblotting analysis.

a, Source figure for **Fig. 2b**. **b,** Source figure for **Supplementary Fig. 6d**. **c,** Source figure for **Supplementary Fig. 8c**.

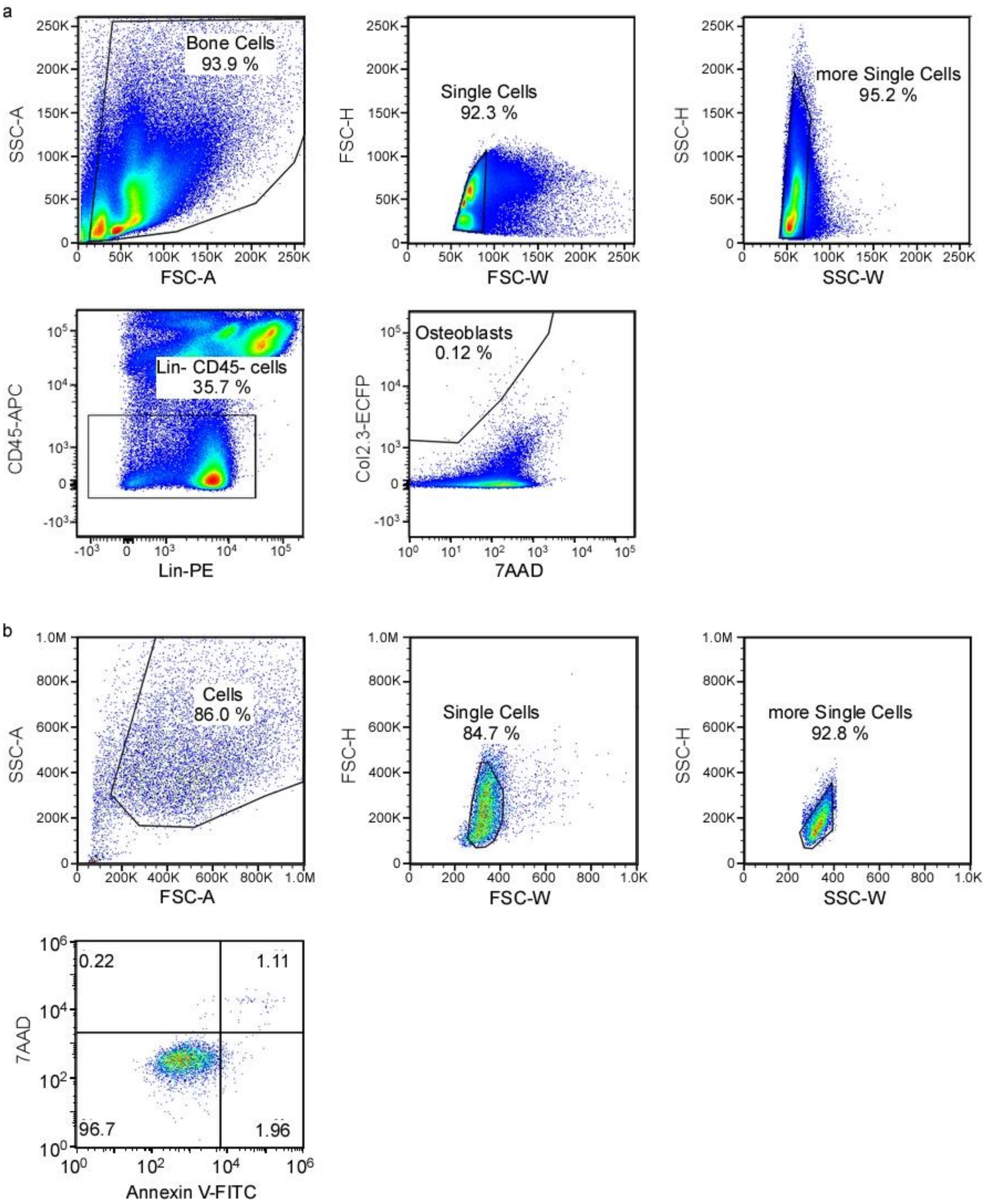

Supplementary Figure 12. FACS gating strategy

a, Gating strategies for ECFP+ osteoblasts (**Supplementary Fig. 1c**). **b,** Gating strategies for the evaluation of apoptotic MC3T3-E1 cells (**Supplementary Fig. 6f**).

Twelve-week-old female mice with intermittent vehicle or PTH for 4 weeks							
Parameters	WT Vehicle (N = 6 mice)	WT PTH (N = 5 mice)	*Slpi*-KO Vehicle (N = 6 mice)	*Slpi*-KO PTH (N = 5 mice)	ANOVA		
					WT Vehicle vs PTH	*Slpi*-KO Vehicle vs PTH	Interaction between *Slpi*-KO and PTH
BV/TV (%)	7.3 ± 0.697	10.7 ± 1.301	5.80 ± 0.771	6.11 ± 0.187	p = 0.0251*	p = 0.9506	p = 0.0962
Tb.Th (µm)	33.5 ± 1.70	39.7 ± 1.27	31.6 ± 2.45	31.3 ± 0.335	p = 0.0427*	p = 0.9954	p = 0.0823
Tb.N (/mm)	2.20 ± 0.115	2.68 ± 0.319	1.79 ± 0.111	1.95 ± 0.072	p = 0.1228	p = 0.7689	p = 0.3627
Tb.Sp (µm)	426 ± 25	356 ± 49	537 ± 40	483 ± 18	p = 0.3130	p = 0.4917	p = 0.8191
MAR (µm/day)	1.66 ± 0.231	2.59 ± 0.123	1.59 ± 0.221	1.81 ± 0.191	p = 0.0220*	p = 0.8718	p = 0.0977
MS/BS (%)	26.1 ± 1.83	37.9 ± 3.30	30.4 ± 1.86	34.5 ± 3.02	p = 0.0173*	p = 0.6506	p = 0.1421
BFR / BS (µm3/µm2/day)	0.161 ± 0.067	0.361 ± 0.096	0.170 ± 0.038	0.230 ± 0.083	p = 0.0002***	p = 0.1899	p = 0.0366*
Ob.S/BS (%)	12.6 ± 4.98	19.0 ± 4.90	8.38 ± 1.95	15.6 ± 1.67	p = 0.0564	p = 0.0366*	p = 0.8098
OS/BS (%)	12.5 ± 1.38	27.5 ± 4.56	7.31 ± 1.98	18.9 ± 7.12	p = 0.0340*	p = 0.1123	p = 0.6767
OV/BV (%)	2.28 ± 0.546	5.46 ± 0.295	1.53 ± 0.454	2.59 ± 1.44	p = 0.0319*	p = 0.5916	p = 0.2089
O.Th (µm)	2.08 ± 0.343	2.66 ± 0.154	1.34 ± 0.362	1.65 ± 0.467	p = 0.4352	p = 0.7848	p = 0.6103
Oc.S/BS (%)	4.52 ± 0.462	5.80 ± 0.795	6.39 ± 0.904	5.64 ± 1.03	p = 0.5401	p = 0.9940	p = 0.5343
ES/BS (%)	9.8 ± 0.87	10.1 ± 1.31	12.9 ± 1.68	12.5 ± 1.31	p = 0.9837	p = 0.9497	p = 0.9244

Supplementary Table 1. Bone histomorphometric analysis of toluidine blue-stained tibial proximal metaphyses from wild-type or *Slpi*-KO mice after PTH treatment.

BFR/BS, bone formation rate per bone surface; BV/TV, bone volume per total volume; ES/BS, eroded surface per bone surface; MAR, mineral apposition rate; MS/BS, mineralizing surface per bone surface; O.Th, osteoid thickness; OS/BS, osteoid surface per bone surface; OV/BV, osteoid volume per bone volume;

Ob.S/BS, osteoblast surface per bone surface; Oc.S/BS, osteoclast surface per bone surface; PTH, parathyroid hormone; Slpi-KO, secretory leukocyte protease inhibitor knockout; Tb.N, trabecular number; Tb.Sp, trabecular separation; Tb.Th, trabecular thickness; WT, wild-type.

Data are mean ± SEM. *; $p < 0.05$, **; $p < 0.01$. Statistical significance was determined by ANOVA with Šidák's test.

Gene	Forward	Reverse
Slpi	CAAACCAGTGTGGAGGAAGC	GAGCAGGGAAGTAGTTTCCAG
Runx2	CGACAGTCCCAACTTCCTGT	CGGTAACCACAGTCCCATCT
Sp7	CATCTGCCTGACTCCTTGGGAC	GCTGAAAGGTCAGCGTATGGC
Col1a1	TGGTTCTCCTGGTTCTCCTG	GCTGAAGTCATAACCGCCAC
Bglap	GGGCAATAAGGTAGTGAACAG	GCAGCACAGGTCCTAAATAGT
Fgf2	GGCTGCTGGCTTCTAAGTGT	CCGTTTTGGATCCGAGTTTA
Ctnnb1	CTGCTCATCCCACTAATGTC	CTTTATTAACTACCACCTGGTCCT
Icam1	CAATTTCTCATGCCGCACAG	AGCTGGAAGATCGAAAGTCCG
Gapdh	ACCACAGTCCATGCCATCAC	TCCACCACCCTGTTGCTGTA

Supplementary Table 2. Primers used for qPCR analyses are listed along with the corresponding gene. Secretory leukocyte protease inhibitor (*Slpi*), runt-related transcription factor 2 (*Runx2*), Sp7 transcription factor (*Sp7*), collagen, type I, alpha 1 (*Col1a1*), bone gamma carboxyglutamate protein (*Bglap*), Fibroblast growth factor 2 (*Fgf2*), catenin (cadherin associated protein), beta 1 (*Ctnnb1*), intercellular adhesion molecule 1 (*Icam1*), glyceraldehyde-3-phosphate dehydrogenase (*Gapdh*).
