# Supplementary material for: SLPI is a critical mediator that controls PTH-induced bone formation
Source: Nat Commun. 2021 Apr 9;12:2136. doi: 10.1038/s41467-021-22402-x (PMC8035405; doi:10.1038/s41467-021-22402-x)
Supplement: Supplementary file 8 — Reporting Summary [file 41467_2021_22402_MOESM8_ESM.pdf]

nature research

Corresponding author(s): DBPR

Last updated by author(s): Jan 16, 2021

Reporting Summary

Nature Research wishes to improve the reproducibility of the work that we publish. This form provides structure for consistency and transparency in reporting. For further information on Nature Research policies, see Authors & Referees and the Editorial Policy Checklist .

Statistics

For all statistical analyses, confirm that the following items are present in the figure legend, table legend, main text, or Methods section.

n/a	Confirmed	
☐	☒	The exact sample size (*n*) for each experimental group/condition, given as a discrete number and unit of measurement
☐	☒	A statement on whether measurements were taken from distinct samples or whether the same sample was measured repeatedly
☐	☒	The statistical test(s) used AND whether they are one- or two-sided<br>*Only common tests should be described solely by name; describe more complex techniques in the Methods section.*
☒	☐	A description of all covariates tested
☐	☒	A description of any assumptions or corrections, such as tests of normality and adjustment for multiple comparisons
☐	☒	A full description of the statistical parameters including central tendency (e.g. means) or other basic estimates (e.g. regression coefficient) AND variation (e.g. standard deviation) or associated estimates of uncertainty (e.g. confidence intervals)
☐	☒	For null hypothesis testing, the test statistic (e.g. *F*, *t*, *r*) with confidence intervals, effect sizes, degrees of freedom and *P* value noted<br>*Give P values as exact values whenever suitable.*
☒	☐	For Bayesian analysis, information on the choice of priors and Markov chain Monte Carlo settings
☒	☐	For hierarchical and complex designs, identification of the appropriate level for tests and full reporting of outcomes
☒	☐	Estimates of effect sizes (e.g. Cohen's *d*, Pearson's *r*), indicating how they were calculated

Our web collection on statistics for biologists contains articles on many of the points above.

Software and code

Policy information about availability of computer code

Data collection	The data were collected using FlowJo 10.4, TRI/3D-BON, Illumina Casava, TopHat, Bowtie2, SAMtools as described in Methods section.
Data analysis	The data were analyzed using Cuffnorm v. 2.2.1, Ingenuity Pathway Software Spring 2020, Pathway Analysis (iDEP) v. 0.81, GraphPad PRISM 9, JMP Pro 15, NIS-Elements 4.50.00, Imaris 9.3.1, R software (ver. 3.6.0), EBImage package (ver. 4.20.0) as described in Methods section.

For manuscripts utilizing custom algorithms or software that are central to the research but not yet described in published literature, software must be made available to editors/reviewers. We strongly encourage code deposition in a community repository (e.g. GitHub). See the Nature Research guidelines for submitting code & software for further information.

Data

Policy information about availability of data

All manuscripts must include a data availability statement. This statement should provide the following information, where applicable:

- Accession codes, unique identifiers, or web links for publicly available datasets
- A list of figures that have associated raw data
- A description of any restrictions on data availability

The datasets are available from the corresponding authors on reasonable request. Raw RNA-Seq data is available from the Gene Expression Omnibus (accession number and hyperlinks: GSE145462 [https://www.ncbi.nlm.nih.gov/geo/query/acc.cgi?acc=GSE145462]).

Field-specific reporting

Please select the one below that is the best fit for your research. If you are not sure, read the appropriate sections before making your selection.

Life sciences study design

All studies must disclose on these points even when the disclosure is negative.

Sample size	All experiments were performed with at least 3 independent biological replicates per condition. Data are displayed as mean ± standard error of the mean (SEM). Statistical analysis was performed using unpaired two-tailed t-tests for comparisons between two groups, and using analysis of variance (ANOVA) with the Dunnett's, Tukey's, and Šidák's post hoc test for comparisons among three or more groups. Welch's ANOVA followed by Bonferroni's multiple comparisons test was used when the data demonstrated unequal variance on GraphPad Prism Software and JMP Software as appropriate and indicated in applicable figure legends. The exact numbers of samples are indicated on scatter dot plots in each figure.
Data exclusions	No samples or animals were excluded from the analysis except for mouse bone histomorphometric analysis (ES/BS, OcS/BS, ObS/BS) where outliers were detected prior to the analysis with Smirnov-Grubbs' test for outliers.
Replication	All western blotting experiments were confirmed at least once with an independent experiment. Experiments using MC3T3-E1 cell line were performed at least three times, but they were not biologically independent because of the homogeneity of this cell line. The rest of the data was obtained from at least three biologically independent experiments. All attempts at replication were successful.
Randomization	For in vivo studies, mice were randomly distributed into treatment groups within each genotype. For in vitro studies, conditions were randomized into control and experimental conditions.
Blinding	The majority of assays were not relevant to blinding, because the treatment groups needed to be clear when performing the experiments in in vitro and in vivo experiments. For the data collection with manual procedures, investigators were blinded to the identity of the experimental group. For bone histomorphometric analysis, investigators were not blinded, because this experiment was from outside orders, and we told the technicians the groups for the proper sample preparation.

Reporting for specific materials, systems and methods

We require information from authors about some types of materials, experimental systems and methods used in many studies. Here, indicate whether each material, system or method listed is relevant to your study. If you are not sure if a list item applies to your research, read the appropriate section before selecting a response.

Materials & experimental systems

n/a	Involved in the study	
☐	☒	Antibodies
☐	☒	Eukaryotic cell lines
☒	☐	Palaeontology
☐	☒	Animals and other organisms
☒	☐	Human research participants
☒	☐	Clinical data

Methods

n/a	Involved in the study	
☒	☐	ChIP-seq
☐	☒	Flow cytometry
☒	☐	MRI-based neuroimaging

Antibodies

Antibodies used

The antibodies used in this study were as follows: anti-CD16/43 antibody (553141, BD Biosciences), anti-CD45-APC (103112, BioLegend), anti-CD45-FITC (103108, BioLegend), anti-CD31-FITC (102405, BioLegend), anti-F4/80-PE/Cy7 (123113, BioLegend), anti-Ly6C-APC (560595, BD Biosciences), anti-Ly6G-BV421 (127627, BioLegend), anti-Sca1-APC (108111, BioLegend), anti-CD51-PE (104105, BioLegend), Streptavidin-PE (405203, BioLegend), Streptavidin-FITC (405201, BioLegend), Annexin V-FITC (640905, BioLegend), 7AAD (559525, BD Pharmingen), Mouse SLPI antibody (AF1735, RSD), β-catenin antibody (610153, BD Biosciences), anti-β-actin-HRP (ab20272, Abcam), anti-ROCK1 antibody (ab134181, Abcam), anti-ROCK2 (ab125025, Abcam), SLPI antibody (AF1274, R&D Systems), SLPI antibody(S0635, US Biological Life Sciences), goat IgG (sc2028, Santa Cruz Biotechnology), goat anti-mouse IgG-HRP (sc-2005, Santa Cruz Biotechnology), rabbit anti-goat IgG-HRP (sc-2768, Santa Cruz Biotechnology)

Validation

All the antibodies used were commercially available. The species and quality validation performed by each manufacturer is described below:

anti-CD16/32 antibody (553141, BD Biosciences)
https://www.bdbiosciences.com/ds/pm/tds/553141.pdf

anti-CD45-APC (103112, BioLegend)
https://www.biolegend.com/en-us/global-elements/pdf-popup/apc-anti-mouse-cd45-antibody-97?filename=APC%20anti-mouse%20CD45%20Antibody.pdf&pdfgen=true

anti-CD45-FITC (103108, BioLegend)
https://www.biolegend.com/en-us/global-elements/pdf-popup/fitc-anti-mouse-cd45-antibody-99?filename=FITC%20anti-mouse%20CD45%20Antibody.pdf&pdfgen=true

anti-CD31-FITC (102405, BioLegend)
https://www.biolegend.com/en-us/global-elements/pdf-popup/fitc-anti-mouse-cd31-antibody-120?filename=FITC%20anti-mouse%20CD31%20Antibody.pdf&pdfgen=true

anti-F4/80-PE/Cy7 (123113, BioLegend)
https://www.biolegend.com/Default.aspx?ID=147&ProductID=4070&filename=PECy7%20anti-mouse%20F480%20Antibody.pdf&pdfgen=true

anti-Ly6C-APC (560595, BD Biosciences)
https://www.bdbiosciences.com/ds/pm/tds/560595.pdf

anti-Ly6G-BV421 (127627, BioLegend)
https://www.biolegend.com/en-us/global-elements/pdf-popup/brilliant-violet-421-anti-mouse-ly-6g-antibody-7161?filename=Brilliant%20Violet%20421trade%20anti-mouse%20Ly-6G%20Antibody.pdf&pdfgen=true

anti-Sca1-APC (108111, BioLegend)
https://www.biolegend.com/en-us/global-elements/pdf-popup/apc-anti-mouse-ly-6a-e-sca-1-antibody-225?filename=APC%20anti-mouse%20Ly-6AE%20Sca-1%20Antibody.pdf&pdfgen=true

anti-CD51-PE (104105, BioLegend)
https://www.biolegend.com/en-us/global-elements/pdf-popup/pe-anti-mouse-cd51-antibody-488?filename=PE%20anti-mouse%20CD51%20Antibody.pdf&pdfgen=true

Streptavidin-PE (405203, BioLegend)
https://www.biolegend.com/en-us/global-elements/pdf-popup/pe-streptavidin-1475?filename=PE%20Streptavidin.pdf&pdfgen=true

Streptavidin-FITC (405201, BioLegend)
https://www.biolegend.com/en-us/global-elements/pdf-popup/fitc-streptavidin-1473?filename=FITC%20Streptavidin.pdf&pdfgen=true

Annexin V-FITC (640905, BioLegend)
https://www.biolegend.com/en-us/global-elements/pdf-popup/fitc-annexin-v-5161?filename=FITC%20Annexin%20V.pdf&pdfgen=true

7AAD (559525, BD Pharmingen)
https://www.bdbiosciences.com/ds/pm/tds/559925.pdf

Rabbit anti-SLPI antibody (NBP1-76396, Novus Biologicals)
https://www.novusbio.com/PDFs/NBP1-76396.pdf

Mouse SLPI antibody (AF1735, RSD)
https://resources.rndsystems.com/pdfs/datasheets/af1735.pdf

β-catenin antibody (610153, BD Biosciences)
https://www.bdbiosciences.com/ds/pm/tds/610153.pdf

anti-β-actin-HRP (ab20272, Abcam)
https://www.abcam.co.jp/beta-actin-antibody-mabcam-8226-loading-control-hrp-ab20272.pdf

anti-ROCK1 antibody (ab134181, Abcam)
https://www.abcam.co.jp/rock1-antibody-epr638y-ab134181.pdf

anti-ROCK2 (ab125025, Abcam)
https://www.abcam.co.jp/rock2-antibody-epr7141b-ab125025.pdf

SLPI antibody (AF1274, R&D Systems)
https://www.rndsystems.com/products/human-slpi-antibody_af1274#product-datasheets

SLPI antibody(S0635, US Biological Life Sciences)
https://www.usbio.net/antibodies/S0635/Secretory%20Leukocyte%20Protease%20Inhibitor/data-sheet

goat IgG (sc2028, Santa Cruz Biotechnology)
https://datasheets.scbt.com/sc-2028.pdf

	goat anti-mouse IgG-HRP (sc-2005, Santa Cruz Biotechnology)<br>https://datasheets.scbt.com/sc-2005.pdf<br><br>rabbit anti-goat IgG-HRP (sc-2768, Santa Cruz Biotechnology)<br>https://datasheets.scbt.com/sc-2768.pdf

Eukaryotic cell lines

Policy information about cell lines

Cell line source(s)	MC3T3-E1 (ATCC) , Plat-E (Cell Biolabs), HEK293T (ATCC)
Authentication	Not authenticated.
Mycoplasma contamination	Not tested.
Commonly misidentified lines<br>(See ICLAC register)	No commonly misidentified lines was used in this study.

Animals and other organisms

Policy information about studies involving animals; ARRIVE guidelines recommended for reporting animal research

Laboratory animals	All mice used in this study were derived on C57BL/6 (B6) background.<br>Slpi-KO mice were provided by A. Nakamura's laboratory (Tohoku Medical and Pharmaceutical University).<br>Slpi-KO mice were bred with Col2.3-ECFP mice and TRAP-tdTomato mice by us to generate Slpi-KO/Col2.3-ECFP/TRAP-tdTomato mice.<br>For most of the studies, 8-16 week old female mice were used.
Wild animals	No wild animal was used in this study.
Field-collected samples	No field-collected sample was used in this study.
Ethics oversight	All animal experiments were performed in accordance with experimental animal guidelines of Osaka University under approved protocols.

Note that full information on the approval of the study protocol must also be provided in the manuscript.

Flow Cytometry

Plots

Confirm that:

- [x] The axis labels state the marker and fluorochrome used (e.g. CD4-FITC).
- [x] The axis scales are clearly visible. Include numbers along axes only for bottom left plot of group (a 'group' is an analysis of identical markers).
- [x] All plots are contour plots with outliers or pseudocolor plots.
- [x] A numerical value for number of cells or percentage (with statistics) is provided.

Methodology

Sample preparation	Calvaria, humerus, ulnas/radius, hips, tibia, and femur bones were crushed, then washed with Hank's balanced salt solution (HBSS). Bone tips were incubated in a 3 mg/mL solution of type I collagenase (Worthington) at 37°C for 25 min. This incubation in collagenase solution was repeated twice (digestions 1–3). Next, bone pieces were incubated in 5 mM ethylenediaminetetraacetic acid (EDTA) solution (digestion 4). Digestions 1–4 were subjected to flow cytometry for isolation of homatopoietic cells, osteoblasts, and MSCs. For in vitro studies, cells were detached from the dishes with trypsin, centrifuged, and collected. Single-cell suspensions were blocked with anti-CD16/32 antibody (BD Biosciences) for 10 min, followed by staining using flow cytometry (FACS) buffer (1× PBS, 4% heat-inactivated FCS, and 2 mM EDTA) for 15 min.
Instrument	FACS Aria II (BD Biosciences) for ECFP+ osteoblast sorting (Supplementary Fig. 1c). SH800 cell sorter (Sony) for the evaluation of MC3T3-E1 cell apoptosis (Supplementary Fig. 6f).
Software	FlowJo software (TreeStar)
Cell population abundance	After sorting CD45-Lin-ECFP+ cell populations, the sorted cells were passed through the cell sorter machine in order to determine the percentage of positive cells. The percentage of ECFP+cell populations was 70 %.
Gating strategy	Preliminary gates were set to exclude cell debris and RBC on FSC/SSC plots. Then single cells were gated on the FSC-H/FSC-W plots. Gating was determined by blank and single cell color-staining. FACS strategies are provided in detail in the Supplementary

Figure 1, 4, and 12.

- [x] Tick this box to confirm that a figure exemplifying the gating strategy is provided in the Supplementary Information.
