# Supplementary material for: SLPI is a critical mediator that controls PTH-induced bone formation
Source: Nat Commun. 2021 Apr 9;12:2136. doi: 10.1038/s41467-021-22402-x (PMC8035405; doi:10.1038/s41467-021-22402-x)
Supplement: Supplementary file 9 — Description of Additional Supplementary Files [file 41467_2021_22402_MOESM9_ESM.pdf]

Title: Supplementary Movie 1
Description: Representative micro-computed tomography images of the femurs of 11-week-old WT female mice treated with vehicle. Scrolling goes through the XY slice-stack. Scale bar, 1,000 µm.

Title: Supplementary Movie 2
Description: Representative micro-computed tomography images of the femurs of 11-week-old WT female mice treated with PTH. Scrolling goes through the XY slice-stack. Scale bar, 1,000 µm.

Title: Supplementary Movie 3
Description: Representative micro-computed tomography images of the femurs of 11-week-old Slpi-KO female mice treated with vehicle. Scrolling goes through the XY slice-stack. Scale bar, 1,000 µm.

Title: Supplementary Movie 4
Description: Representative micro-computed tomography images of the femurs of 11-week-old Slpi-KO female mice treated with PTH. Scrolling goes through the XY slice-stack. Scale bar, 1,000 µm.

Title: Supplementary Movie 5
Description: In vitro imaging of mock MC3T3-E1-EGFP cells in contact with TRAP-tdTomato+ primary osteoclasts. Filled arrowheads indicate direct osteoblast–osteoclast contact. Scale bar: 50 µm. Playback speed = 1,440×.

Title: Supplementary Movie 6
Description: In vitro imaging of Slpi-overexpressing MC3T3-E1- EGFP cells in contact with TRAP-tdTomato+ primary osteoclasts. Filled arrowheads indicate direct osteoblast–osteoclast contact. Scale bar: 50 µm. Playback speed = 1,440×
